# Integrating feature selection with unsupervised deep embedding for clustering single-cell RNA-seq data

**DOI:** 10.1093/bib/bbag082

**Published:** 2026-03-02

**Authors:** Cheng Zhong, Siqi Jiang, Zhi Wei

**Affiliations:** Department of Computer Science, New Jersey Institute of Technology, 323 Dr Martin Luther King Jr Blvd, Newark, NJ 07102, United States; Department of Computer Science, New Jersey Institute of Technology, 323 Dr Martin Luther King Jr Blvd, Newark, NJ 07102, United States; Department of Computer Science, New Jersey Institute of Technology, 323 Dr Martin Luther King Jr Blvd, Newark, NJ 07102, United States

**Keywords:** scRNA-seq, dimension reduction, group lasso, deep learning

## Abstract

Single-cell RNA sequencing (scRNA-seq) enables high-resolution analysis of gene expression at the individual cell level, with clustering serving as a critical step for identifying distinct cell populations. Due to the high dimensionality and sparsity of scRNA-seq data, existing approaches typically perform gene selection prior to clustering. However, treating feature selection as a separate preprocessing step can overlook latent clustering structure and often results in suboptimal outcomes, as it does not guarantee that the selected genes are informative for clustering. To address this limitation, we propose **FSSC** (Feature Selection for scRNA-seq Clustering), a unified framework for joint feature selection and clustering in scRNA-seq analysis. FSSC integrates a zero-inflated negative binomial (ZINB) autoencoder with a group Lasso penalty and a dedicated clustering loss. This joint optimization enables the model to simultaneously learn low-dimensional representations and select a compact set of cluster-discriminatory genes, preserving both the statistical characteristics of scRNA-seq data and its underlying cluster structure. Extensive experiments on both simulated and real scRNA-seq datasets demonstrate that FSSC consistently outperforms state-of-the-art methods in clustering accuracy and effectively identifies a compact, biologically meaningful set of marker genes.

## Introduction

Single-cell RNA sequencing (scRNA-seq) technology provides detailed information of gene expression at the individual cell level and has profoundly changed the paradigm of biomedical research [1]. The scRNA-seq data is commonly represented as a high-dimensional sparse matrix, where each row corresponds to a cell and each column corresponds to a gene (feature). The discrete count matrix output from scRNA-seq exhibits extremely high variance. Furthermore, the amount of RNA obtained from each single cell is small while the sequencing depth per cell is shallow, which results in the generation of many ‘false’ zero values in the count matrix. This is referred to as the dropout event, where more than 50% of observations can be zero [2]. The high levels of noise and sparsity pose significant challenges in the analysis of scRNA-seq data. Clustering cells into different groups is an essential step in many scRNA-seq studies, as it can provide researchers with important insights into cell heterogeneity within the data. To address these analytic and computational challenges, many clustering methods have been proposed, including hierarchical clustering [3], kernel-based spectral clustering [4, 5], and deep learning-based clustering [6–10].

Typical scRNA-seq datasets profile thousands to tens of thousands of genes across the transcriptome. After clustering cells, differential expression (DE) analysis is often used to identify genes with significant expression differences across clusters, aiding in biological interpretation. However, clustering performance and interpretability are often hindered by the high-dimensional feature space. Moreover, DE analysis frequently reveals that many genes are uninformative for distinguishing cell types, underscoring the need for effective feature (gene) selection. Existing strategies usually perform selection prior to clustering, with Seurat [11] selecting highly variable genes (HVGs), and NBDrop [12] retaining genes with high dropout rates. Yet these methods are clustering-agnostic and may not capture the true cluster structure. Moreover, improper feature selection can lead to false discovery of subpopulations through excessive subdivision of homogeneous cell clusters [13].

To address this, earlier efforts from the microarray era introduced feature selection into clustering, particularly for ‘high dimension, low sample size’ settings. For example, Pan et al. [14] proposed a penalized model-based clustering approach that modeled microarray gene expression data with a finite Normal mixture model, penalized mean parameters with an L1 penalty, and derived an EM algorithm with a modified BIC for model selection. As RNA-seq replaced microarray as the standard platform for transcriptomics, gene expression began to be quantified by gene-level read counts, which are discrete and therefore violate Normal assumptions. FSCseq [15] addressed this by modeling RNA-seq count data using a mixture of multivariate negative binomial (NB) regression models. It applied a SCAD fusion penalty [16] on pairwise differences between log2 cluster means to perform feature selection, and optimized the penalized log-likelihood using a CEM algorithm with simulated annealing.

Compared with bulk RNA-seq, scRNA-seq count data are even sparser due to dropout events. To address the zero-inflation problem, RZiMM [17] extended penalized mixture models with zero-inflated distributions, using a Gaussian model for normalized data and Poisson or NB models for raw counts. Like FSCseq, it applies a similar fusion penalty but replaces the SCAD penalty with an L1 penalty to penalize mean parameters.

However, existing penalized mixture models treat each gene independently and aim to identify marker genes associated with cell clusters, disregarding gene–gene dependencies. This gene-wise treatment often results in highly redundant gene lists and may increase experimental validation costs without improving biological insight. Recent self-supervised learning approaches have shown promise in capturing feature representations for cell clustering [18], yet they do not explicitly address feature selection and gene redundancy. In contrast, identifying a compact, nonredundant gene set that sufficiently explains cluster structure can improve interpretability, reduce costs, and enhance computational efficiency and model generalization.

The effectiveness of deep learning in scRNA-seq clustering and the value of feature selection motivate their integration. In supervised settings, methods such as Sparse-input Neural Networks [19] and LassoNet [20] apply group Lasso [21] to select informative features in high-dimensional data. In contrast, unsupervised methods like Concrete Autoencoder [22] focus on reconstruction rather than clustering, and are not tailored for identifying cluster-discriminative features. Additionally, while some recent methods have incorporated contrastive learning for improving cell annotation [23], they typically require pre-existing annotations and do not directly perform feature selection in fully unsupervised settings.

To address the limitations of existing methods in clustering and feature selection for scRNA-seq data, we propose **F**eature **S**election for **s**cRNA-seq **C**lustering approach (FSSC). FSSC integrates a zero-inflated negative binomial (ZINB)-based autoencoder with a group Lasso-regularized input layer to simultaneously address dropout-induced sparsity and identify cluster-discriminative, nonredundant genes. The ZINB component captures the zero-inflation characteristic of scRNA-seq data and learns informative low-dimensional embeddings for clustering, while the group Lasso penalty encourages sparse feature selection and accounts for gene dependencies—overcoming the limitations of conventional filtering or penalized models that treat genes independently. Furthermore, since ground-truth labels are unavailable in unsupervised settings, FSSC introduces a model selection strategy that adaptively tunes the regularization parameter by assessing clustering stability across iterations, enabling robust and principled feature selection without supervision.

## Method

### Problem formulation

Let $\boldsymbol{X}$ ∈ ${\mathbb{R}}^{N\times G}$ represent the $N\times G$ gene read count matrix of $N$ cells and $G$ features (genes). We aim to learn a subset of features $S$ ⊆ {1, 2, …, $G$}, where $\left|S\right|$< $G$ and a reconstruction function $f$, such that the difference between the reconstructed features $f$ (${\boldsymbol{X}}_S$) and the original features $\boldsymbol{X}$ is minimized and the clustering performance based on the ${\boldsymbol{X}}_S$ is optimized. Namely, we want to optimize:


(1) \begin{equation*} \underset{S,f}{\mathrm{argmin}}\ {\mathcal{L}}_R\left(f\left({\boldsymbol{X}}_S\right),\boldsymbol{X}\right)+\gamma{\mathcal{L}}_C\left({\boldsymbol{X}}_S\right) \end{equation*}

where ${\mathcal{L}}_R$ and ${\mathcal{L}}_C$ are the loss functions to evaluate the reconstruction difference and the clustering performance, and the hyperparameter $\gamma$ > 0 balances the relative weights of the two losses.

**Figure 1:**
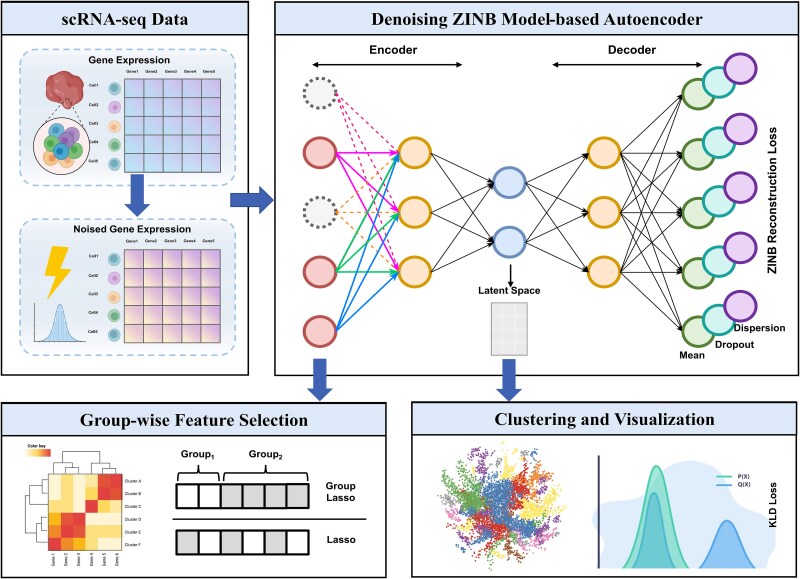
The encoder and decoder are composed of fully connected neural networks. A reconstruction loss based on a ZINB model quantifies the difference between the reconstructed and original gene expression profiles. Clustering is performed in the learned latent space. To enforce sparsity and identify informative genes, a group lasso penalty is applied to the weights of the encoder’s first hidden layer.

The core idea is that a small set of truly discriminatory features should suffice to reconstruct the original data and recover the underlying cluster structure. To enforce this, we build an autoencoder that learns to reconstruct its input, but we add a group-Lasso penalty on the weights of the encoder’s first hidden layer so that only the most informative features survive. We then run clustering on the resulting low-dimensional embedding. Figure 1 illustrates the full architecture.

Let $g$ be the decoder and we overload ${f}_W$ as the encoder with weights $W$, where ${W}^{(1)}$ represents the weights of the first hidden layer of the encoder and ${W}_{\cdotp j}^{(1)}$ denotes the weights for the $j$th feature in ${W}^{(1)}$. If $\parallel{W}_{\cdotp j}^{(1)}\parallel$= 0, the $j$th feature is not selected in the subset $S$. The objective function of the proposed model FSSC is defined as:


(2) \begin{equation*} \underset{S,f,g}{\mathrm{argmin}}\ {\mathcal{L}}_R(g({f}_W(\boldsymbol{X})),\boldsymbol{X})+\gamma{\mathcal{L}}_C({f}_W(\boldsymbol{X}))+\lambda{\sum}_{j=1}^G\kern0.1em {\left\| {W}_{\cdotp j}^{(1)}\right\|}_2 \end{equation*}

where $\lambda$ is the group Lasso penalty parameter, which controls the sparsity of the model. The higher values of $\lambda$, the fewer features are selected.

### Data simulation

To evaluate the performance of FSSC in clustering and feature selection, we conducted the following simulation studies. We first applied the R package Splatter [24] to simulate sRNA-seq count data for 4000 cells of 10 000 genes from four approximately equal-sized groups (around 1000 cells per group). We then sampled 1000 of the 10 000 genes to generate a 4000 $\times$ 1000 raw count matrix in the following way. We divided the 10 000 genes into discriminatory ${G}_d$ and nondiscriminatory genes ${G}_{nd}$, where nondiscriminatory genes are genes with DE factor’s values (DEFacGroup value generated by the Splatter) equal to 1 for all groups, and discriminatory genes, otherwise. To simulate data with different signal strengths, we selected a subset of ${G}_d$ by filtering the genes with $de{f}_{\mathrm{min}}\le \mid$ DEFacGroup $\mid \le de{f}_{\mathrm{max}}$. We next applied R package glmnet [25] to remove highly correlated genes in the selected subsets by training a multinomial classification model with a group Lasso penalty, where the model inputs are the selected gene subsets and the outputs are the ground truth cell assignments. Let ${r}_{dg}$ be the proportion of discriminatory genes among 1000 genes. We adjusted the Lasso penalty to keep $1000\cdotp{r}_{dg}$ discriminatory genes derived from the glmnet, sampled $1000\left(1-{r}_{dg}\right)$ nondiscriminatory genes from ${G}_{nd}$ and concatenated them to generate the final simulation dataset. We repeated all experiments 10 times under the same setting. The detailed simulation settings are summarized in Supplementary Note S4.

### Competing methods

We compared FSSC with six competing methods: scDeepCluster [8], Seurat [11], Scvis [10]+k-means, SC3 [26], FSCseq [15] and RZiMM-NB [17]. The first four are clustering-only methods, while FSCseq and RZiMM-NB perform clustering and feature selection simultaneously. Therefore, clustering performance was evaluated against all six methods. Feature selection was further compared among FSCseq, RZiMM-NB and three gene selection methods, Seurat, NBDrop [12]and FEAST [27]. Seurat selects high-variance genes, NBDrop identifies genes with high dropout rates, and FEAST ranks genes using F-statistics across clusters.

We used clustering accuracy and Adjusted Rand Index (ARI) [28] to assess clustering performance, where higher values indicate better results. For gene selection, FSCseq outputs binary selections, while RZiMM-NB, Seurat, NBDrop, and FEAST provide gene rankings. An additional threshold is required to select genes; for NBDrop, we used a significance threshold of 0.05. Precision and Recall were used to evaluate FSCseq and NBDrop, while AUC was computed for RZiMM-NB, Seurat, NBDrop, and FEAST.

The detailed implementation and parameter settings of FSSC and competing methods are summarized in Supplementary Note S2 and Supplementary Note S3, respectively.

### Denoising ZINB model-based autoencoder

We employ a denoising autoencoder to learn a low-dimensional embedding of the preprocessed read-count matrix (Supplementary Note S1) to a low-dimensional embedding space for clustering and map it back to the original count matrix. Let $\overset{\sim }{\boldsymbol{X}}$ ∈ ${\mathbb{R}}^{N\times G}$ represent the $N\times G$ preprocessed read count matrix of $N$ cells and $G$ features. The input for the denoising autoencoder is ${\overset{\sim }{\boldsymbol{X}}}^{\mathrm{corrupt}}$ = $\overset{\sim }{\boldsymbol{X}}+\boldsymbol{e}$, where $\boldsymbol{e}$ is the Gaussian noise.

Let $Z={f}_W\left({\overset{\sim }{\boldsymbol{X}}}^{\mathrm{corrupt}}\right)$ and $g(Z)$ denote the low-dimensional embedding generated by the encoder and the output of the decoder, respectively. Both encoder and decoder are multi-layer fully connected neural networks with RELU activation function [29]. The learning process of the denoising autoencoder is to minimize the loss function $\mathcal{L}\left(\boldsymbol{X},g\left({f}_W\left({\overset{\sim }{\boldsymbol{X}}}^{\mathrm{corrupt}}\right)\right)\right)$.

We then apply a ZINB model-based reconstruction loss to model the high dropout event in the scRNA-seq data. This loss function uses the likelihood of a ZINB distribution to characterize scRNA-seq data. Let ${X}_{ig}^{\mathrm{count}}$ denote the raw read count of cell $i$ and gene $g$. The ZINB distribution is parameterized with mean ${\mu}_{ig}$, dispersion ${\theta}_{ig}$, and an additional coefficient ${\pi}_{ig}$ that represents the dropout probability:


(3) \begin{equation*} NB\left({X}_{ig}^{\mathrm{count}}\mid{\mu}_{ig},{\theta}_{ig}\right)=\frac{\Gamma \left({X}_{ig}^{\mathrm{count}}+{\theta}_{ig}\right)}{X_{ig}^{\mathrm{count}}!}{\left(\frac{\theta_{ig}}{\theta_{ig}+{\mu}_{ig}}\right)}^{\theta_{ig}}{\left(\frac{\theta_{ig}}{\theta_{ig}+{\mu}_{ig}}\right)}^{X_{ig}^{\mathrm{count}}} \end{equation*}

(4) \begin{align*} &ZINB\left({X}_{ig}^{\mathrm{count}}\mid{\pi}_{ig},{\mu}_{ig},{\theta}_{ig}\right)\nonumber \\ &\quad ={\pi}_{ig}{\delta}_0\left({X}_{ig}^{\mathrm{count}}\right)+\left(1-{\pi}_{ig}\right) NB\left({X}_{ig}^{\mathrm{count}}\mid{\mu}_{ig},{\theta}_{ig}\right) \end{align*}

We append three independents fully connected layers ${g}_{\mu }$, ${g}_{\theta }$ and ${g}_{\pi }$ to the last layer of the decoder $g$ to estimate the ZINB parameters


(5) \begin{equation*} {\mu}_i=\mathrm{diag}\left({s}_i\right)\times \mathrm{exp} \left({g}_{\mu}\left(g\left({\mathbf{Z}}_i\right)\right)\right) \end{equation*}

(6) \begin{equation*} {\theta}_{\boldsymbol{i}}=\mathrm{softplus}\left({g}_{\theta}\left(g\left({\mathbf{Z}}_i\right)\right)\right) \end{equation*}

(7) \begin{equation*} {\pi}_{\boldsymbol{i}}=\mathrm{sigmoid}\left({g}_{\pi}\left(g\left({\mathbf{Z}}_i\right)\right)\right) \end{equation*}

where $\mu$, $\theta$, and $\pi$ represent the matrix form of the estimated mean, dispersion, and dropout probability, respectively. ${s}_i$ is the precalculated library size factor of cell $i$ (Supplementary Note S1) and included as an independent input to the model. The loss function of the ZINB model-based autoencoder is the sum of the negative log of the ZINB likelihood:


(8) \begin{equation*} {\mathcal{L}}_{ZINB}=-\sum_{i,g}\kern0.1em \log \left( ZINB\left({X}_{ig}^{\mathrm{count}}\mid{\pi}_{ig},{\mu}_{ig},{\theta}_{ig}\right)\right) \end{equation*}

### Clustering on the latent embedding space

We employ a self-training [30] strategy to implement clustering on the latent embedding space [8, 31, 32]. Let $Z={f}_W\big(\overset{\sim }{\boldsymbol{X}}\big)$ be the low-dimensional latent representation generated by the encoder. Let $Q$ be the distribution of soft labels measured by Student’s t-distribution, and $P$ be the derived target distribution from $Q$. The clustering loss function is defined as the Kullback–Leibler (KL) divergence between $P$ and $Q$:


(9) \begin{equation*} {\mathcal{L}}_C= KL\left(P\parallel Q\right)=\sum_{i=1}^N\kern0.1em \sum_{j=1}^C\kern0.1em {p}_{ij}\, \mathrm{log} \frac{p_{ij}}{q_{ij}} \end{equation*}

where $C$ is the number of clusters and ${q}_{ij}$ is the soft label assignment of embedding ${z}_i$ defined by the similarities between the embedding ${z}_i$ and clustering centroid ${\mu}_j$ calculated by the Student’s t-distribution:


(10) \begin{equation*} {q}_{ij}=\frac{{\left(1+{\parallel{z}_i-{\mu}_j\parallel}^2\right)}^{-1}}{\sum_{j^{\prime }}\kern0.20em {\left(1+{\parallel{z}_i-{\mu}_{j^{\prime }}\parallel}^2\right)}^{-1}} \end{equation*}

and ${p}_{ij}$ is the target distribution applied in the self-training:


(11) \begin{equation*} {p}_{ij}=\frac{q_{ij}^2/\sum_i\kern0.20em {q}_{ij}}{\sum_{j^{\prime }}\kern0.20em \left({q}_{i{j}^{\prime}}^2/\sum_i\kern0.20em {q}_{i{j}^{\prime }}\right)} \end{equation*}

The target distribution $Q$ is built based on $P$. Furthermore, at each iteration, minimizing the clustering loss function ${\mathcal{L}}_C$ will push $Q$ toward the target distribution $P$.

Thus, the loss function of our model is calculated as:


(12) \begin{equation*} \mathcal{L}={\mathcal{L}}_{ZINB}+\gamma{\mathcal{L}}_C \end{equation*}

where ${\mathcal{L}}_{ZINB}$ and ${\mathcal{L}}_C$ are the ZINB-based reconstruction loss of the autoencoder and the clustering loss, respectively. The hyperparameter $\gamma$ > 0 controls the relative weights of the two losses.

### Feature selection with group lasso penalty

Our method performs the clustering and feature selection simultaneously by adding a group Lasso penalty on the weights associated with the first hidden layer of the encoder. The objective function in Equation (2) can be rewritten as follows:


(13) \begin{equation*} \underset{W^{(1)},U}{\mathrm{argmin}}\ \mathcal{L}\left(\boldsymbol{X}\right)+\lambda \sum_{j=1}^G\kern0.1em {\left\| {W}_{\cdotp j}^{(1)}\right\|}_2 \end{equation*}

where $\mathcal{L}$ is the total loss function, ${W}^{(1)}$ is the weights of the first hidden layer of the encoder, and $U$ represents the weights of the entire neural network except ${W}^{(1)}$.

Building on the Sparse-Group Lasso framework [33], we update the model weights via a proximal-gradient scheme. The full update routine is given in Algorithm 1 (Supplementary Note S7). Concretely, at each iteration $t$, we first carry out a vanilla gradient-descent step on $U$ and ${W}^{(1)}$, then apply a soft-thresholding operator to ${W}^{(1)}$.

### Self-learning model selection strategy

An essential hyperparameter in FSSC is the sparsity-controlling parameter $\lambda$, which determines the strength of the group Lasso penalty. Larger values of $\lambda$ encourage more aggressive feature filtering, resulting in a sparser model. However, determining an appropriate $\lambda$ is particularly challenging in unsupervised deep learning, where classical model selection methods such as cross-validation or BIC are inapplicable or unreliable due to the absence of labels and the model’s nonlinear nature.

To address this, we introduce a self-learning strategy that dynamically adjusts $\lambda$ through a warm-start procedure. We consider a sequence of values ${\lambda}_0=0<{\lambda}_1<\cdots <{\lambda}_T={\lambda}_{\mathrm{max}}$, gradually increasing the sparsity of the network. At each iteration, we train the model using the features selected from the previous $\lambda$ as initialization for the next round, optimizing model parameters through backpropagation.

We begin by training the model with ${\lambda}_0=0$, i.e. without any sparsity constraint, to obtain initial latent embeddings and clustering centroids, using the ZINB reconstruction loss ${\mathcal{L}}_{\mathrm{ZINB}}$ and clustering loss ${\mathcal{L}}_C$ as described in Tian et al. [8]. During subsequent rounds, $\lambda$ is incremented (Algorithm 2, Line11 in Supplementary Note S7), and the group Lasso penalty is applied to remove irrelevant features based on their group norm in the input layer weight matrix ${W}^{(1)}$. After each update, any feature whose corresponding weight vector becomes zero is excluded from future updates by masking its gradient.

To ensure robust clustering and feature selection, we introduce a restart mechanism: after a sufficient number of features have been filtered out, we reinitialize the model and begin a new round of training from scratch (Algorithm 2, Line 9 in Supplementary Note S7). This process is repeated until the number of retained features falls below a user-defined threshold $\xi$.

To determine the optimal $\lambda$, we compare the clustering assignment difference across successive rounds. Specifically, we compute the ARI between predicted cluster assignments from consecutive rounds. Let ${y}_r^{\prime }$ and ${y}_{r-1}^{\prime }$ denote clustering labels at round $r$ and $r-1$ respectively. The ARI between them, denoted as $pAR{I}_r=\mathrm{ARI}({y}_r^{\prime },{y}_{r-1}^{\prime})$, measures the consistency of clustering structure across sparsity levels. To mitigate fluctuations, we apply a five-step moving average to the $pARI$ series to obtain a smoothed sequence $mpAR{I}_r$. We select the final model at the round ${r}^{\ast }$ where $mpAR{I}_r$ is maximized:


(14) \begin{equation*} {r}^{\ast }=\underset{r}{\mathrm{argmax}}\left( mpAR{I}_r\right) \end{equation*}

### Feature importance ranking

In addition to selecting features, FSSC provides a ranking that reflects their relative importance in contributing to cluster separation. This is achieved by analyzing the order in which features are removed as $\lambda$ increases during the training process.

We define a fixed increasing sequence of penalty values ${\lambda}_0,$  ${\lambda}_1,$  $\cdots, {\lambda}_T$, where each ${\lambda}_t$ yields a model with progressively fewer active features. At each step $t$, some features are newly filtered out (i.e. their group norm becomes zero). While multiple features may be removed simultaneously at a given ${\lambda}_t$, we further differentiate among them based on their magnitudes before the thresholding operation. Specifically, we record the ${L}_2$ norm of each feature’s weight vector prior to being zeroed out. Features with larger pre-thresholding norms are considered more important and are ranked higher within that group.

This ranking procedure is carried out at every iteration $t$ and the results are concatenated across all iterations to form a cumulative feature ranking. Let each iteration yield a partial rank list; these are merged in order of removal to produce a final, global ranking that reflects when and how decisively each feature was excluded from the model. If multiple training rounds are used (as described above), this process is repeated independently for each round. The final overall feature ranking is obtained by concatenating the ranked lists across all rounds.

### Data availability

The Kidney dataset was originally published by Adam et al. [34], who performed cell type annotation and transcriptomic profiling of kidney tissue. The preprocessed expression matrix, consisting of 3660 cells from eight annotated cell types, was obtained from https://github.com/xuebaliang/scziDesk.

The Liver and Bladder datasets were generated by Han et al. [35], who characterized single-cell transcriptomes across multiple human tissues, including liver and bladder. We downloaded the batch-corrected expression matrices from https://figshare.com/s/865e694ad06d5857db4b and extracted cells corresponding to liver and bladder tissues.

### Code availability

All the codes are available in the GitHub: https://github.com/sjiang225/FSSC

## Results

### Simulation results

We evaluated FSSC on synthetic datasets to assess its robustness to dropout, varying signal strengths, and discriminatory gene ratios. We also examined the benefits of joint feature selection and clustering compared to decoupled alternatives.

### Robustness to dropout

**Figure 2:**
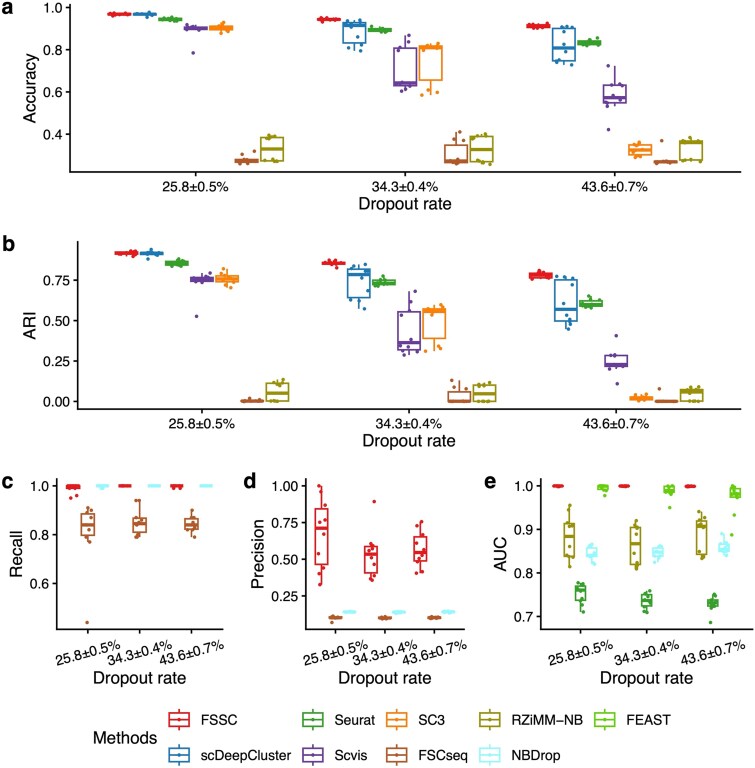
Clustering and gene selection performance on simulated data with various dropout rates. (a)(b): Clustering performance of FSSC, scDeepCluster, Seurat, Scvis, SC3, FSCseq, and RZiMM-NB. (c)(d): Gene selection performance on FSSC, FSCseq, and NBDrop in recall and precision. (e): Gene selection performance on FSSC, RZiMM-NB, Seurat, NBDrop, and FEAST in AUC.

We first examined the impact of increasing dropout levels on clustering and gene selection performance while fixing the ratio of discriminatory genes at ${r}_{dg}=0.1$. By adjusting the midpoint parameter in the logistic dropout function, we generated datasets with mean dropout rates of 25.8%, 34.3%, and 43.6%. As shown in Fig. 2a and b, FSSC consistently outperforms all competing methods in clustering accuracy and ARI, with only modest performance degradation under higher dropout. In contrast, methods such as FSCseq and RZiMM-NB, which are based on rigid mixture models for raw counts, exhibit sharp declines, likely due to their inability to handle zero inflation and overdispersion in scRNA-seq data. Although FSCseq is tailored for RNA-seq, its lack of explicit dropout modeling further limits its performance in single-cell settings.

In gene selection evaluation (Figs 2c–e), FSSC maintains strong and balanced performance across recall, precision, and AUC. While NBDrop achieves similar recall, its low precision suggests over-selection and false positives. FSSC’s higher precision indicates its effectiveness in isolating truly cluster-discriminative genes by jointly optimizing selection and clustering. Compared to Seurat and FEAST, which ignore clustering structure or treat selection as a separate step, FSSC yields more biologically meaningful gene sets. Although RZiMM-NB and FSCseq also aim for integrated modeling, their poor robustness and scalability under high dropout further underscore the stability and interpretability advantages of FSSC in realistic single-cell conditions.

### Varying signal strengths

**Figure 3:**
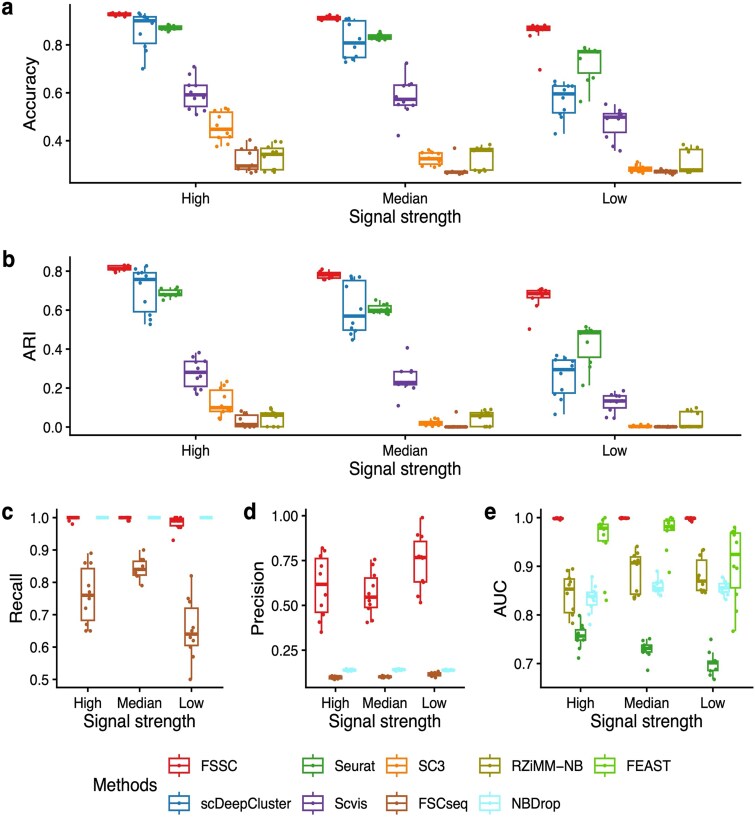
Clustering and gene selection performance on simulated data with various signal strengths. (a)(b): Clustering performance of FSSC, scDeepCluster, Seurat, Scvis, SC3, FSCseq and RZiMM-NB. (c)(d): Gene selection performance on FSSC, FSCseq and NBDrop in recall and precision. (e): Gene selection performance on FSSC, RZiMM-NB, Seurat, NBDrop and FEAST in AUC.

We next evaluated the performance under different signal strengths. We set ${r}_{dg}=0.1$ and generated three settings with different signal strengths by selecting discriminatory genes with different levels of the DE factors. Larger DE factors represent larger signal strength. The experimental results in Fig. 3 reveal that FSSC outperforms other benchmarks in both clustering and feature selection performance.

In particular, we observe that as the signal strength drops, the clustering performance of almost all benchmarks decreases dramatically, while FSSC achieves relatively stable results. It suggests the importance of selecting cluster-discriminatory genes for clustering. Furthermore, the feature selection results illustrated in Figs 3c–e show that FSSC outperforms other benchmarks. These results show the effectiveness of our model in selecting the genes that are informative for clustering.

### Effect of discriminatory gene ratio

We then demonstrate the model performance under the different ratios of discriminatory genes. We fix the dropout rate and change the discriminatory feature ratio ${r}_{dg}\in \left[0.05,\kern0.5em 0.15\right]$. Moreover, we then adjust the signal strength for each ${r}_{dg}$ to control the total signal strength such that the algorithms can achieve approximately the same clustering performance under different settings. As shown in Supplementary Fig. S1, FSSC outperforms all other benchmarks on clustering and feature selection tasks.

### Benefit of joint optimization

To demonstrate the benefit of integrating clustering and feature selection, we first compared FSSC with scDeepCluster using only the ground-truth discriminatory genes across varying signal strengths (Supplementary Fig. S2). Although scDeepCluster performs well with ideal inputs, its accuracy and AUC decline sharply as signal strength weakens. In contrast, FSSC achieves comparable performance without prior knowledge of the true features, highlighting its ability to identify and exploit cluster-discriminatory genes during training. These results emphasize the advantage of embedding feature selection within the clustering process, particularly under low-signal conditions.

We further evaluated FSSC against a two-stage pipeline (AE + Cluster), where feature selection is first performed using a denoising autoencoder with group Lasso, followed by clustering via scDeepCluster (Supplementary Fig. S3). Despite similar gene importance rankings (Fig. S3e), FSSC consistently outperforms AE + Cluster in clustering accuracy and selection precision. AE + Cluster’s performance is hindered by the difficulty of selecting an optimal feature threshold. These findings reinforce the value of FSSC’s joint optimization strategy.

### Feature Selection Path and Adaptive λ Tuning

Supplementary Fig. S4a shows how clustering performance evolves along FSSC’s feature selection path. The number of selected genes decreases as lambda increases, which proves that a larger regularization results in more genes pruning. As less informative genes are pruned, Accuracy and ARI initially improve, plateau, and then decline once truly informative features are removed, confirming the expected trade-off between feature count and clustering quality. Notably, the moving average ARI ($mpARI$) closely tracks this trend and serves as a reliable indicator for selecting the optimal feature subset. The $mpARI$ peak, marked by a vertical dashed line, corresponds to maximal clustering performance and is used to guide final selection. Supplementary Fig. S4b further validates this criterion, showing that at the $mpARI$ peak, FSSC maintains high recall and improved precision, ensuring effective preservation of truly discriminatory genes.

### Ablation on dropout Modeling

To assess the flexibility of FSSC, we conducted an ablation study using synthetic datasets with zero dropout and varying signal strengths. We compared the original ZINB-based model (FSSC(ZINB)) with a variant using a standard Negative Binomial loss (FSSC(NB)), and included FSCseq as a baseline due to its NB-based mixture modeling. As shown in Supplementary Fig. S5, FSSC(ZINB) and FSSC(NB) perform nearly identically and both outperform FSCseq in clustering and feature selection accuracy. These results demonstrate FSSC’s robustness and adaptability, showing that its performance is preserved even when the reconstruction loss is modified.

We further compared the performance of FSSC(ZINB) and FSSC(NB) under different dropout levels using the same simulation setting as in the above dropout experiments. As show in Supplementary Fig. S6, FSSC(ZINB) consistently outperforms FSSC(NB), with significant improvements in ARI (one-sided t-test, *P* < .01) for the dropout rate of 25.8%, 34.3% and 43.6%. The performance gap increases as the dropout level becomes more severe, which proves the advantage of using ZINB loss in high dropout scenarios.

### Ablation on number of clusters

The proposed method relies on prior knowledge of the number of clusters K. Because such prior knowledge may not always be available, we evaluated the model performance when the number of clusters in the dataset and the number of clusters used in the FSSC algorithm is different. Specifically, we simulated data with four clusters and ran the algorithm with K = 4, 6, and 8. As expected, clustering performance decreased when K was mismatched (Supplementary Fig. S7a and b). Nevertheless, the sets of selected genes were generally consistent across these settings (Supplementary Fig. S7c and d), suggesting that the feature selection process is robust to moderate deviations in K.

### Runtime comparison

We evaluated the computational efficiency of FSSC by benchmarking its running time on simulated datasets containing 4000 cells and 1000 genes. As illustrated in Supplementary Fig. S6, FSSC requires more computation time than scDeepCluster, primarily due to the additional overhead of iteratively tuning the sparsity parameter $\lambda$ and performing repeated clustering steps during feature selection. Nonetheless, FSSC is notably more efficient than FSCseq and RZiMM-NB, the two statistical methods that also integrate clustering and feature selection within a unified framework. These results demonstrate that while FSSC introduces moderate computational cost relative to deep learning baselines, it remains computationally competitive compared to existing joint modeling approaches.

### Application to real scRNA-seq data

We applied FSSC to three real scRNA-seq datasets to assess its performance on complex biological systems. Dataset statistics are summarized in Table 1 (Supplementary Note S8), and detailed descriptions are provided in Supplementary Note S5. For all methods, we selected the top 2000 HVGs as input.

### Clustering performance

**Figure 4:**
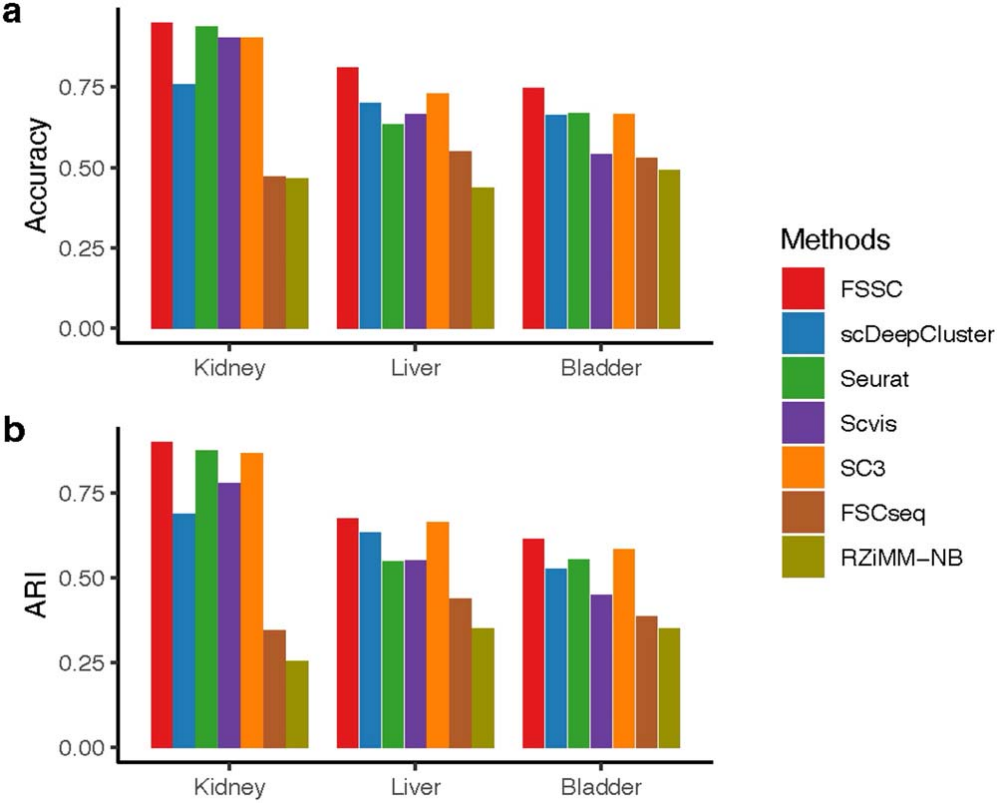
Comparison of clustering performances of FSSC measured by accuracy and ARI.

Figure 4 reports clustering performance across real datasets using Accuracy and ARI. FSSC consistently outperforms all competing methods, demonstrating its robust performance across diverse biological contexts. Notably, although FSCseq and RZiMM-NB are designed to jointly perform clustering and gene selection, they yield the weakest results. This suggests that their reliance on mixture modeling and direct likelihood assumptions limits their effectiveness, particularly when handling complex, high-dimensional single-cell data. In contrast, FSSC leverages deep representation learning together with structured sparsity via group Lasso, enabling it to effectively capture the underlying cluster structure and select informative features, thereby achieving superior clustering accuracy.

### Feature selection evaluation

**Figure 5:**
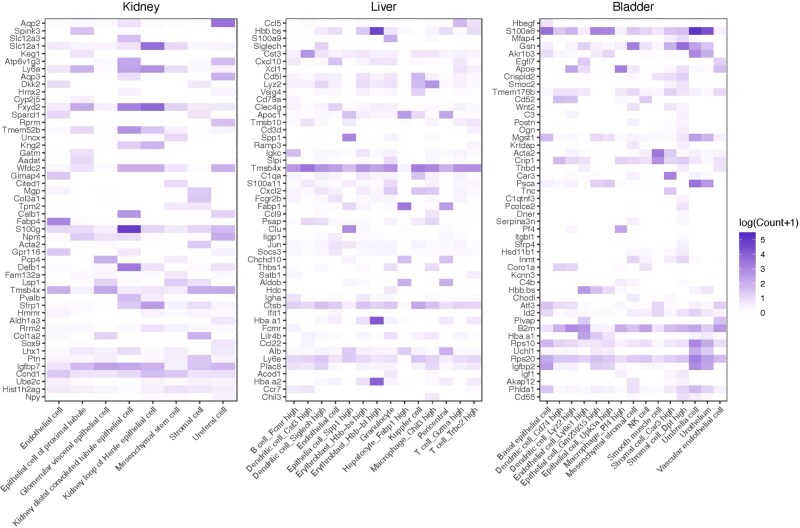
Heatmap of gene expression of the top 50 genes generated by FSSC for the real scRNA-seq dataset.

We compared feature selection performance across FSSC, FSCseq, and NBDrop. As shown in Table 2 (Supplementary Note S8), FSCseq retains over 90% of input genes, offering little dimensionality reduction and limited interpretability. In contrast, FSSC and NBDrop apply more selective filtering. To assess the biological relevance of selected genes, we visualized the top 50 ranked genes from each method on three benchmark datasets. Gene expression was aggregated by ground-truth cell types, log-transformed, and presented as heatmaps (Fig. 5, Supplementary Figs S7–S10), with rows representing genes (ranked by importance) and columns representing cell types.

FSSC-selected genes exhibit pronounced expression differences across cell types, indicating strong cluster specificity. Notably, many of these genes are consistent with established cell-type markers. In the Kidney dataset, FSSC correctly identifies Aqp2, Aqp3, and Aqp4, which are principal cell markers involved in water resorption and electrolyte balance, as well as Osr2, Lhx1, and Jag1, which are markers of early proximal tubule progenitor cells [34]. It also selects Rbp1, Napsa, and Ttc36, known markers of mature proximal tubule cells. Overall, FSSC recovered 17 out of the 41 known marker genes reported in [34] for the kidney dataset (Supplementary Note S8, Table 4), demonstrating the highest marker precision among all compared methods despite selecting far fewer genes. In the Liver dataset, Clec4f and Clec4d are recovered, both of which are established markers of liver-resident macrophages, such as Kupffer cells [35]. Similarly, in the Bladder dataset, stromal cell-specific genes including Wnt2, Cxcl12, Bmp4, and Bmp5 are identified, reflecting tissue-specific expression patterns [35].

**Figure 6:**
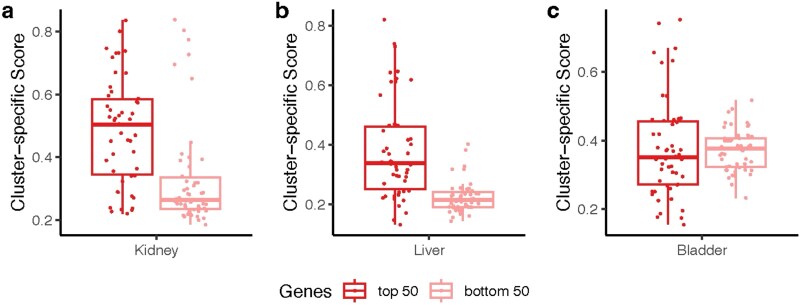
Cluster-specific score for the top and bottom 50 genes generated by FSSC for the real scRNA-seq dataset.

To quantify the informativeness of selected genes, we computed the cluster-specific score [36, 37], an entropy-based metric ranging from 0 to 1 that reflects gene expression specificity across cell types (defined in Supplementary Note S6). As shown in Fig. 6, the top 50 genes selected by FSSC consistently achieve higher scores than the bottom 50 in both Kidney and Liver datasets, confirming its ability to prioritize biologically relevant genes. In the Bladder dataset, the difference is less pronounced, likely due to co-expressed or redundant genes, which may cause the Lasso penalty to retain only representative features.

To demonstrate the biological relevance of the selected genes, we compared FSSC-selected genes with DE genes identified by MAST [38] (FDR < 0.01 and logFC >1). The DE analysis was performed under two labeling settings: (1) using the ground-truth cluster labels, and (2) using the cluster assignments derived from FSSC. The heatmaps of the top 50 genes from each method are shown in Supplementary Figs S13–S15. In addition, we quantified the overlap between the top 100 genes selected by FSSC and those from the two DE analyses in Table 3 (Supplementary Note S8). For example, in the Liver dataset, 45 genes overlapped between FSSC-selected genes and DE genes obtained using the ground-truth labels. To assess whether these overlaps occurred by chance, we conducted a hypergeometric test for each dataset. All overlaps yielded *P* < .01, indicating that the intersection between FSSC-selected genes and standard DE genes is statistically significant and not due to random coincidence. This result supports FSSC’s ability to identify biologically meaningful, differentially expressed genes that contribute effectively to clustering.

We further illustrated the structural advantage of FSSC by visualizing the embeddings of these methods using t-SNE. As shown in Supplementary Fig. S16, the embedding generated by FSSC exhibit separated and compact clusters, where points from the same cluster are tightly grouped together. In contrast embeddings from other methods show more scattered points and some points are mixed between cluster boundaries. It demonstrates that FSSC has the ability to preserve underlying cluster structure in the low-dimensional space.

## Discussion

We proposed FSSC, a model-based deep learning framework that jointly performs feature selection and clustering for scRNA-seq data. By integrating a ZINB-based autoencoder with a group Lasso penalty, FSSC identifies a compact set of informative genes while preserving cluster structure in a low-dimensional space. Extensive experiments on simulated and real datasets demonstrate that FSSC achieves improved clustering accuracy and biologically meaningful gene selection compared to existing methods. In addition, our framework is flexible and can be adapted to other distributions, such as the negative binomial.

Despite its effectiveness, FSSC has several limitations. The performance is sensitive to the sparsity-controlling hyperparameter $\lambda$, and the warm-start strategy for updating $\lambda$ depends on a manually chosen step size $\epsilon$. More adaptive schemes for $\lambda$ selection may improve efficiency and robustness. Moreover, compared to methods like FSCseq that retain correlated marker groups, FSSC may discard redundant yet relevant features due to the nature of Lasso regularization, and future work could explore alternatives such as the Elastic Net [39]. Lastly, FSSC assumes that only a small subset of genes is cluster-discriminatory; its advantage may diminish when many features are relevant or when differences between features are subtle.

**Key Points**

- FSSC integrates a Zero-Inflated Negative Binomial (ZINB) autoencoder with structured sparsity regularization to jointly perform feature selection and clustering for single-cell RNA-seq (scRNA-seq) data, effectively addressing the challenges of high dimensionality, dropout noise, and gene redundancy.
- FSSC enables the identification of biologically meaningful gene subsets by enforcing nonredundant feature selection, which facilitates interpretable clustering and reduces downstream experimental costs in marker gene validation.
- FSSC introduces a self-supervised model selection strategy that dynamically adjusts the sparsity penalty based on clustering stability across iterations, making it robust to the absence of ground-truth labels.
- FSSC achieves state-of-the-art clustering performance and selects compact gene panels on both simulated and real scRNA-seq datasets, demonstrating its practical utility in cell type discovery and biological interpretation.

## Supplementary Material

Supplementary_1024_bbag082
