# Supplementary material for: Integrating feature selection with unsupervised deep embedding for clustering single-cell RNA-seq data
Source: Brief Bioinform. 2026 Mar 2;27(2):bbag082. doi: 10.1093/bib/bbag082 (PMC12951082; doi:10.1093/bib/bbag082)
Supplement: Supplementary_1024_bbag082 [file supplementary_1024_bbag082.docx]

**Supplementary Figures**


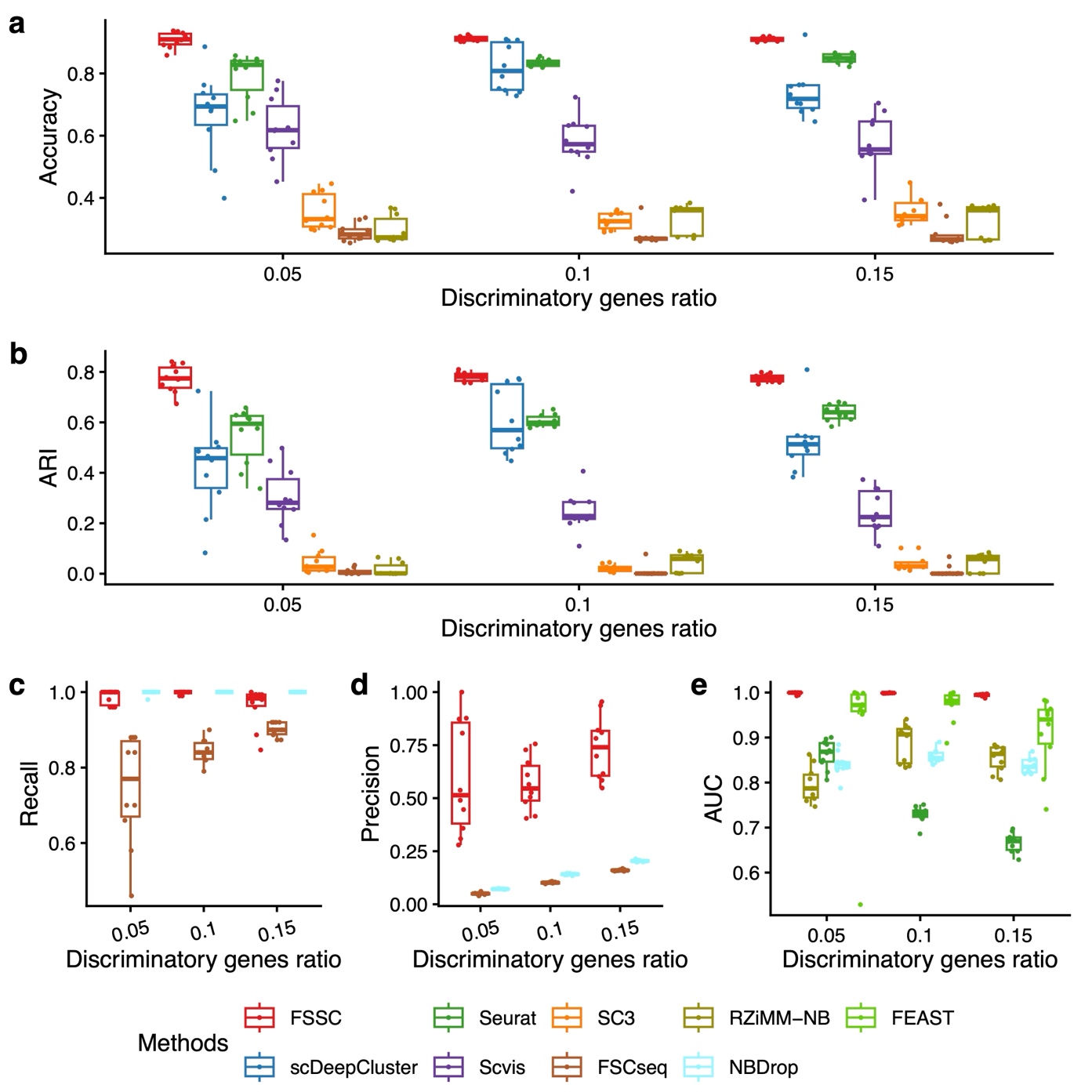


**Supplementary Figure S1.** Clustering and gene selection performance on simulated data with various ratios of discriminatory genes. (**a**)(**b**) Clustering performance of FSSC, scDeepCluster, Seurat, Scvis, SC3, FSCseq, and RZiMM-NB. (**c**)(**d**) Gene selection performance on FSSC, FSCseq and NBDrop in Recall and Precision. (**e**) Gene selection performance on RZiMM-NB, Seurat, NBDrop and FEAST in AUC.


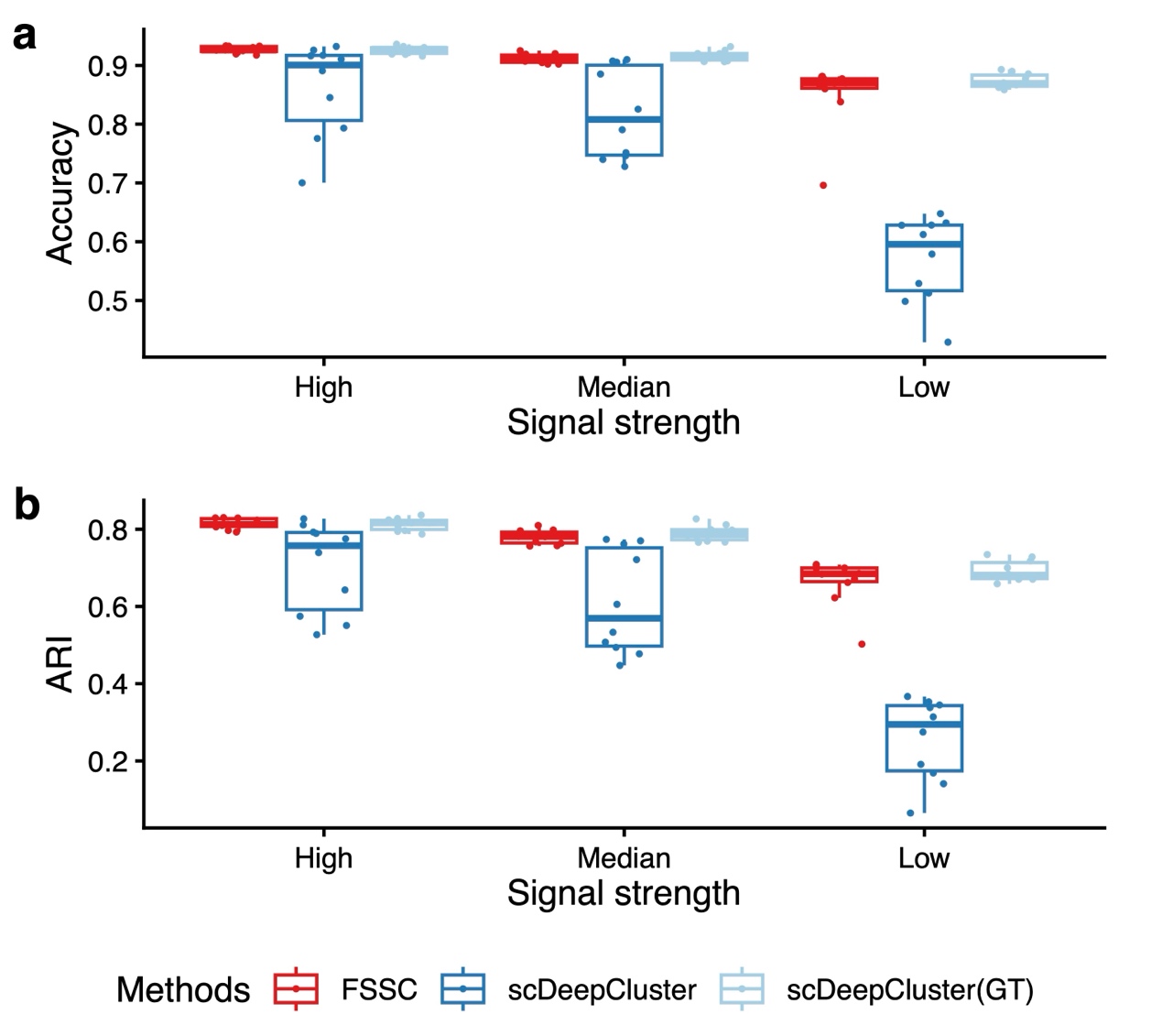


**Supplementary Figure S2.** Clustering performance of FSSC, scDeepCluster, and scDeepcluster using only ground truth features as input.


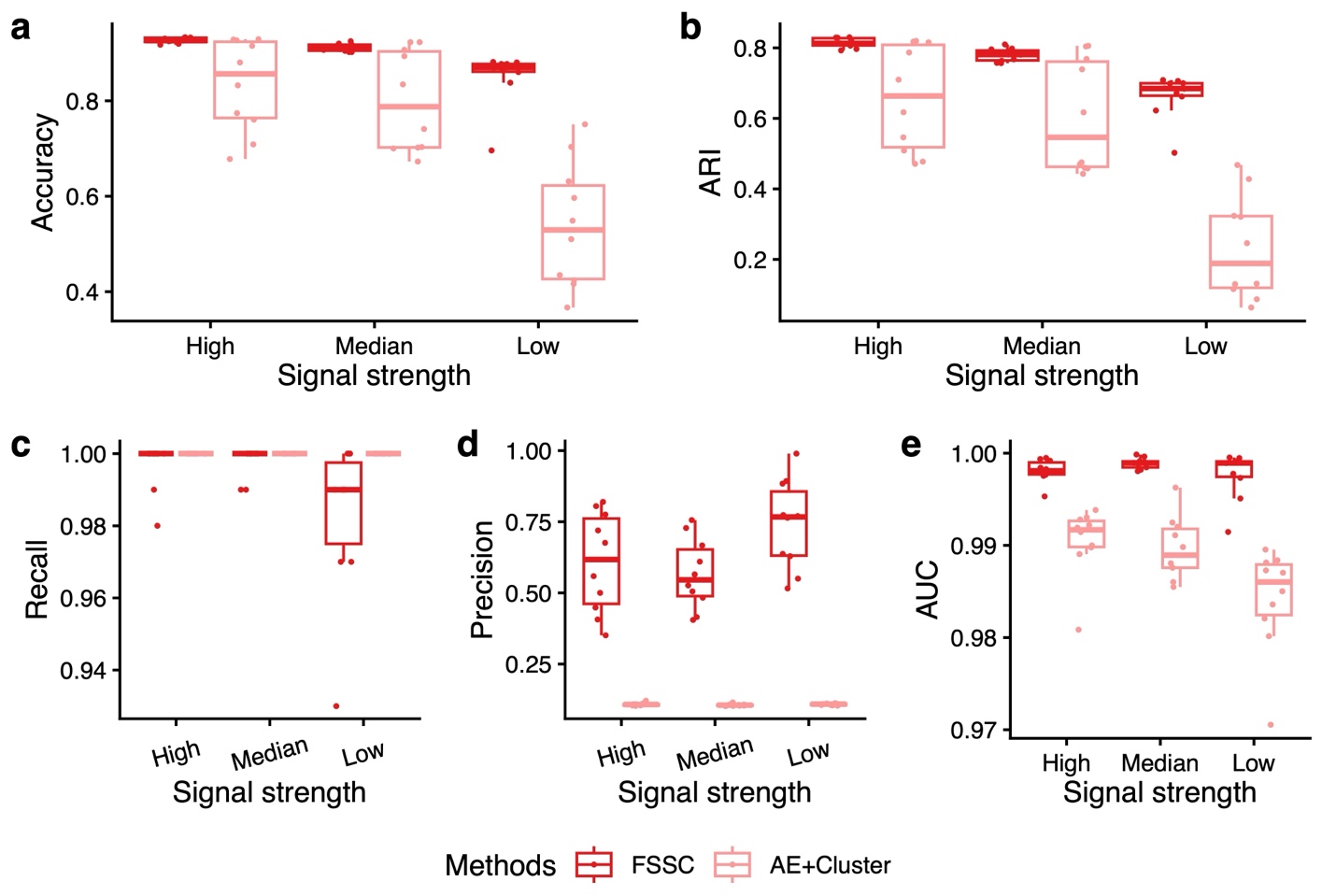


**Supplementary Figure S3.** Clustering and gene selection performance of FSSC and the implementation of feature selection and clustering as separate procedures (AE+Cluster).


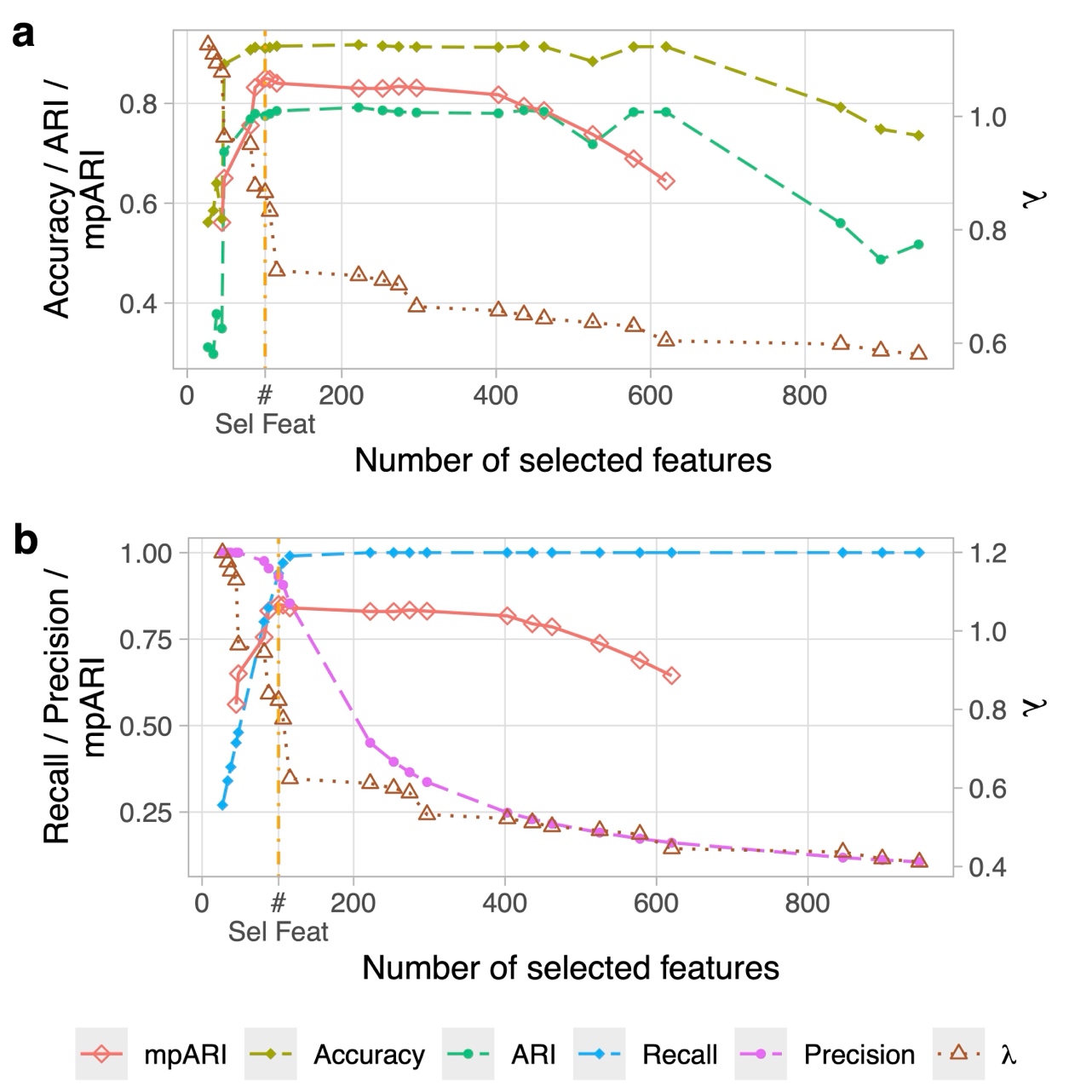


**Supplementary Figure S4.** Clustering performance and feature selection path. (**a**) Clustering performance in Accuracy, ARI, and $mpARI$, with λ shown on the right Y-axis. (**b**) Gene selection performance in Recall, Precision and $mpARI$, with λ shown on the right Y-axis.


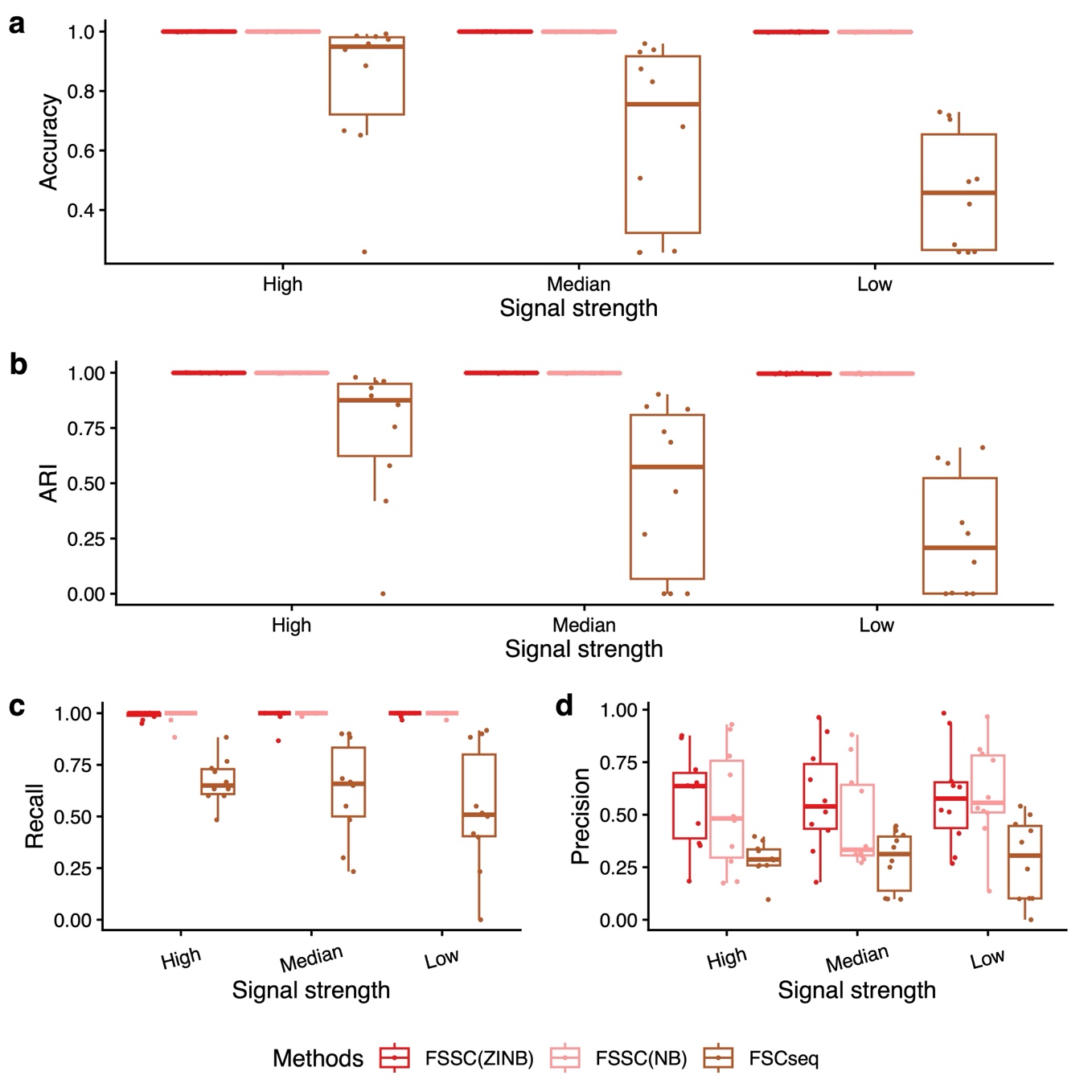


**Supplementary Figure S5.** Clustering and gene selection performance on simulated data without dropout events. Comparison of the performance of FSSC with ZINB loss (FSSC(ZINB)), FSSC with NB loss (FSSC(NB)) and FSCseq.


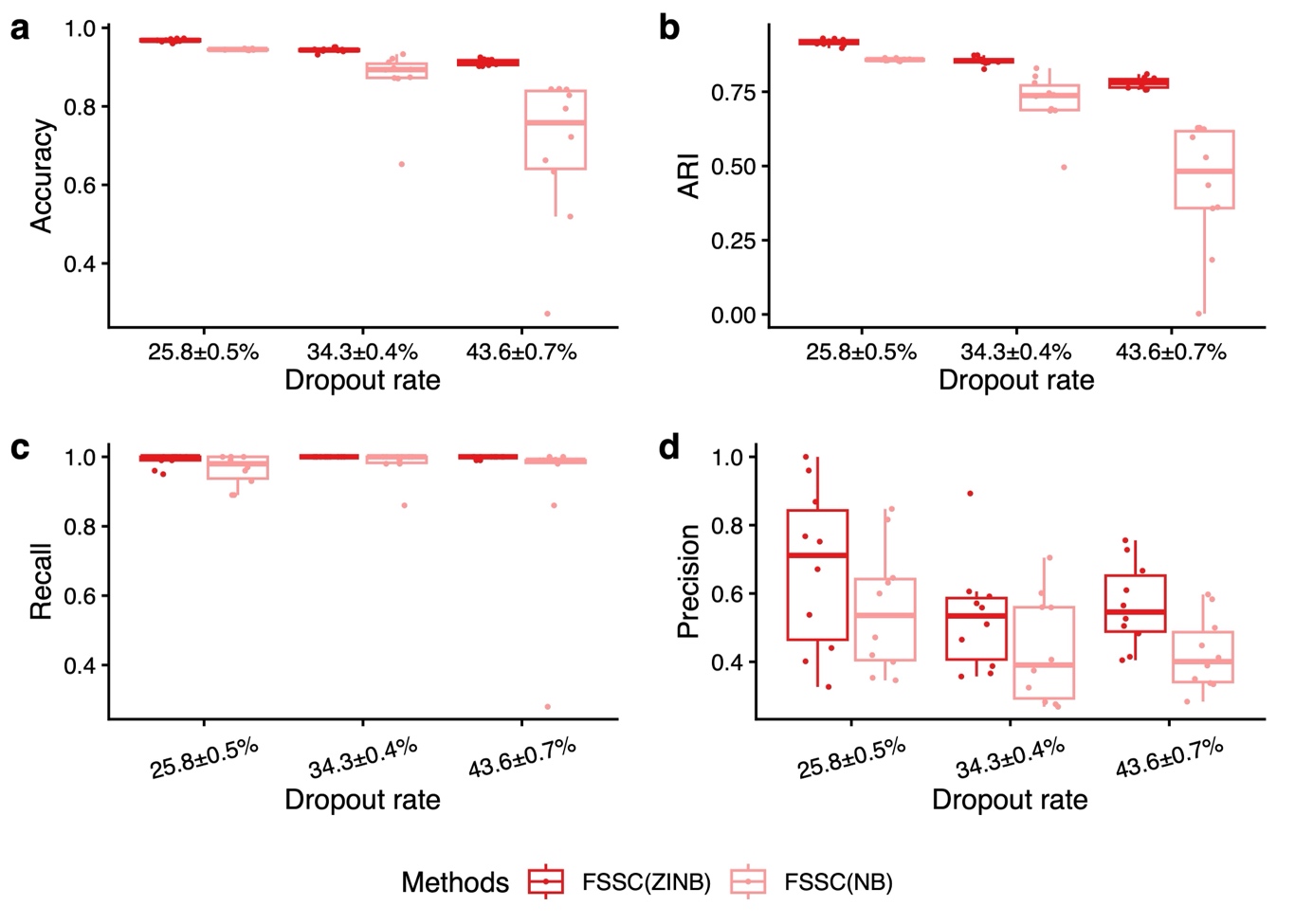


**Supplementary Figure S6.** Clustering and gene selection performance on simulated data with various dropout events. Comparison of the performance of FSSC with ZINB loss (FSSC(ZINB)), FSSC with NB loss (FSSC(NB)).


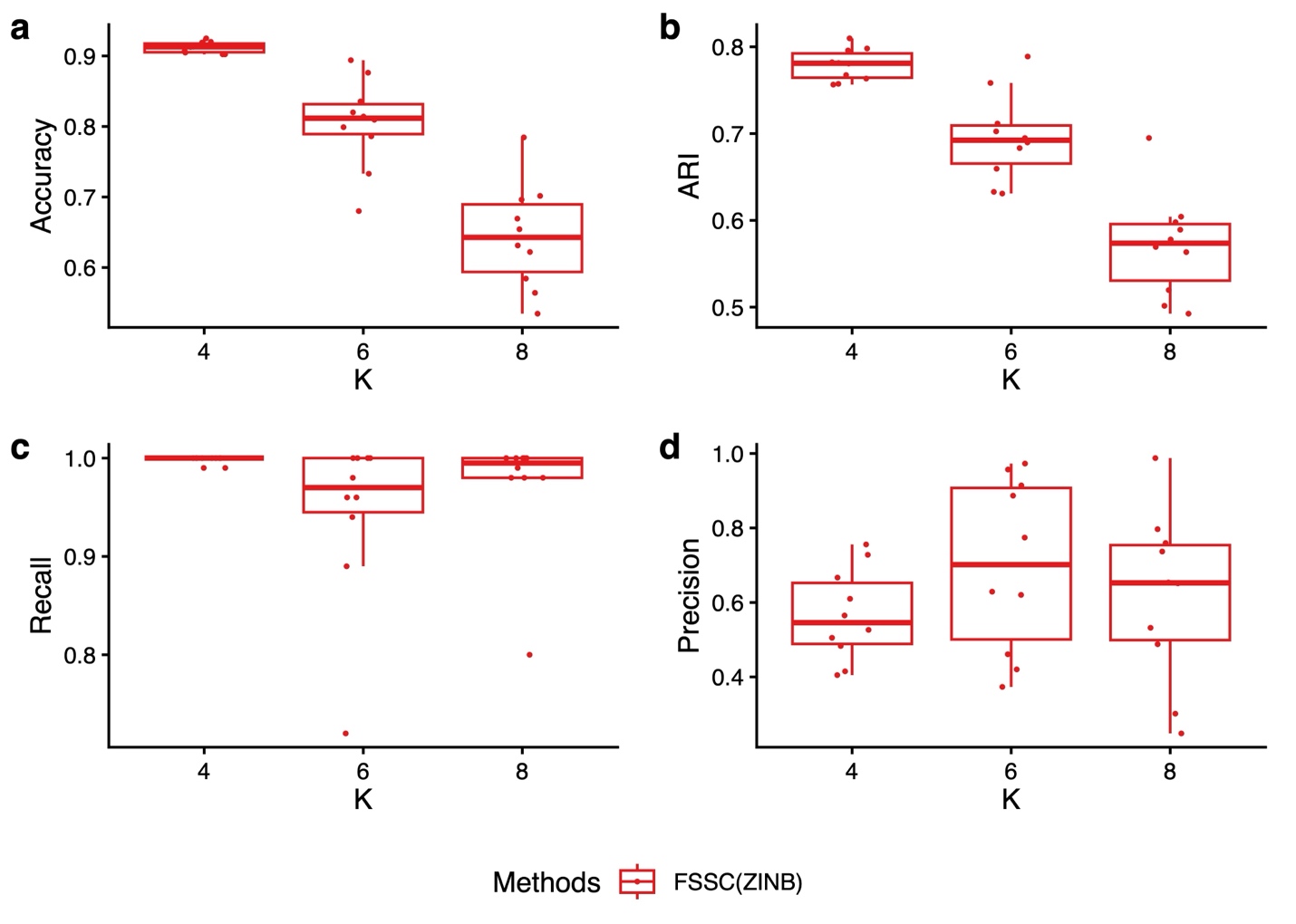


**Supplementary Figure S7.** Clustering and gene selection performance on simulated data. Comparison of FSSC clustering across different numbers of clusters (K = 4, 6, 8)


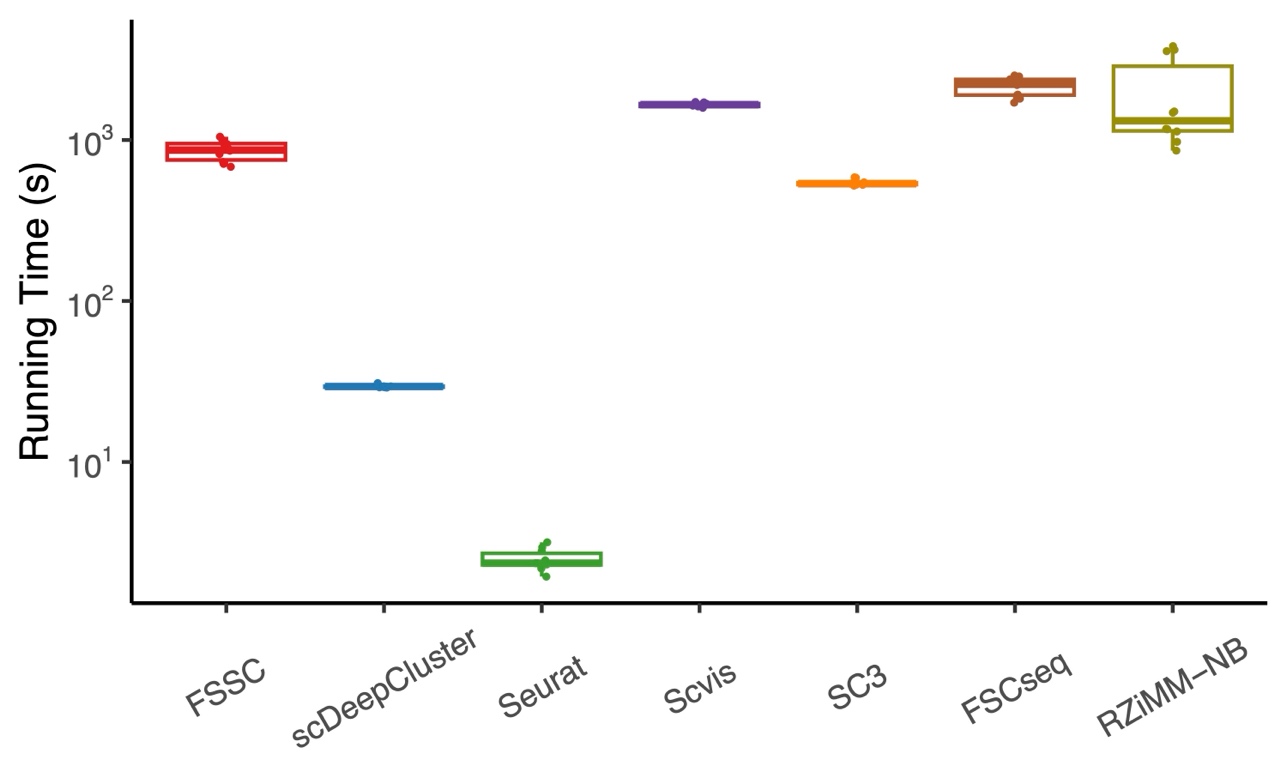


**Supplementary Figure S8.** Comparison of algorithm running time on simulated data. The experiments are conducted on NVIDIA Tesla V100 GPU and Intel Xeon Gold 6248 CPU.


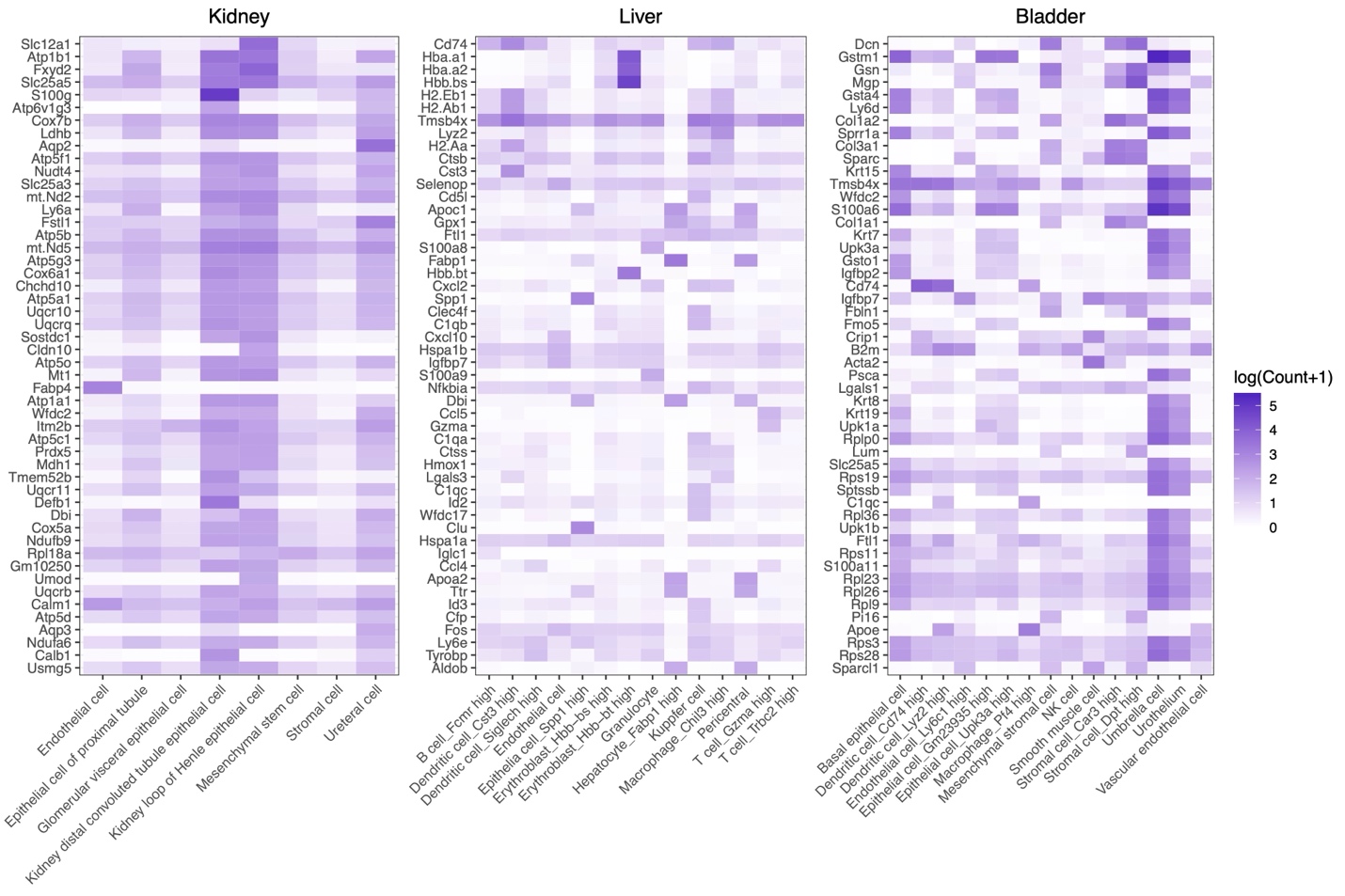


**Supplementary Figure S9.** Heatmap of gene expression of the top 50 genes generated by RZiMM-NB for the real scRNA-seq dataset.

**
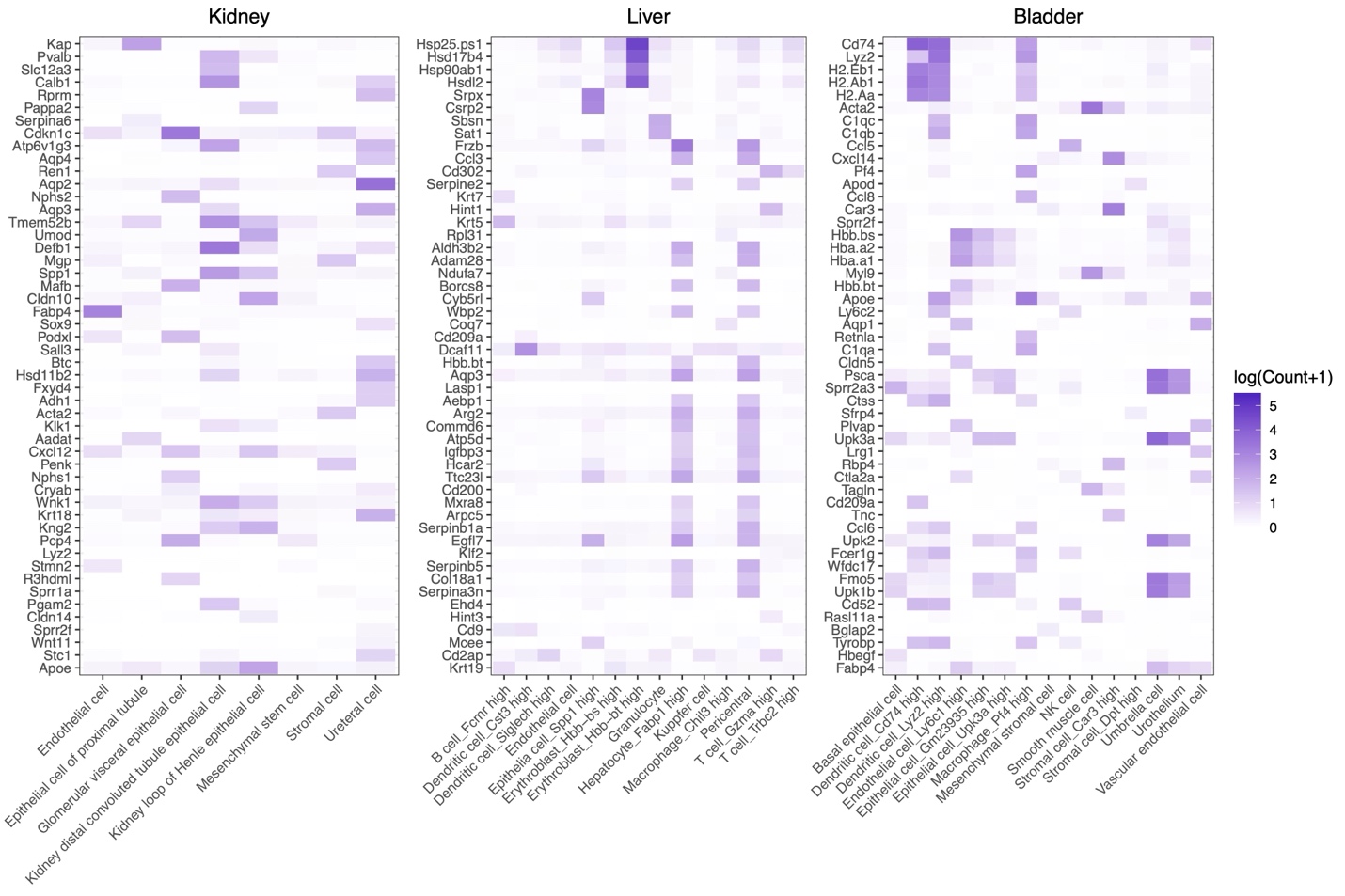
**

**Supplementary Figure S10.** Heatmap of gene expression of the top 50 genes generated by Seurat for the real scRNA-seq dataset.

**Supplementary Figure S11.** Heatmap of gene expression of the top 50 genes generated by NBDrop for the real scRNA-seq dataset.


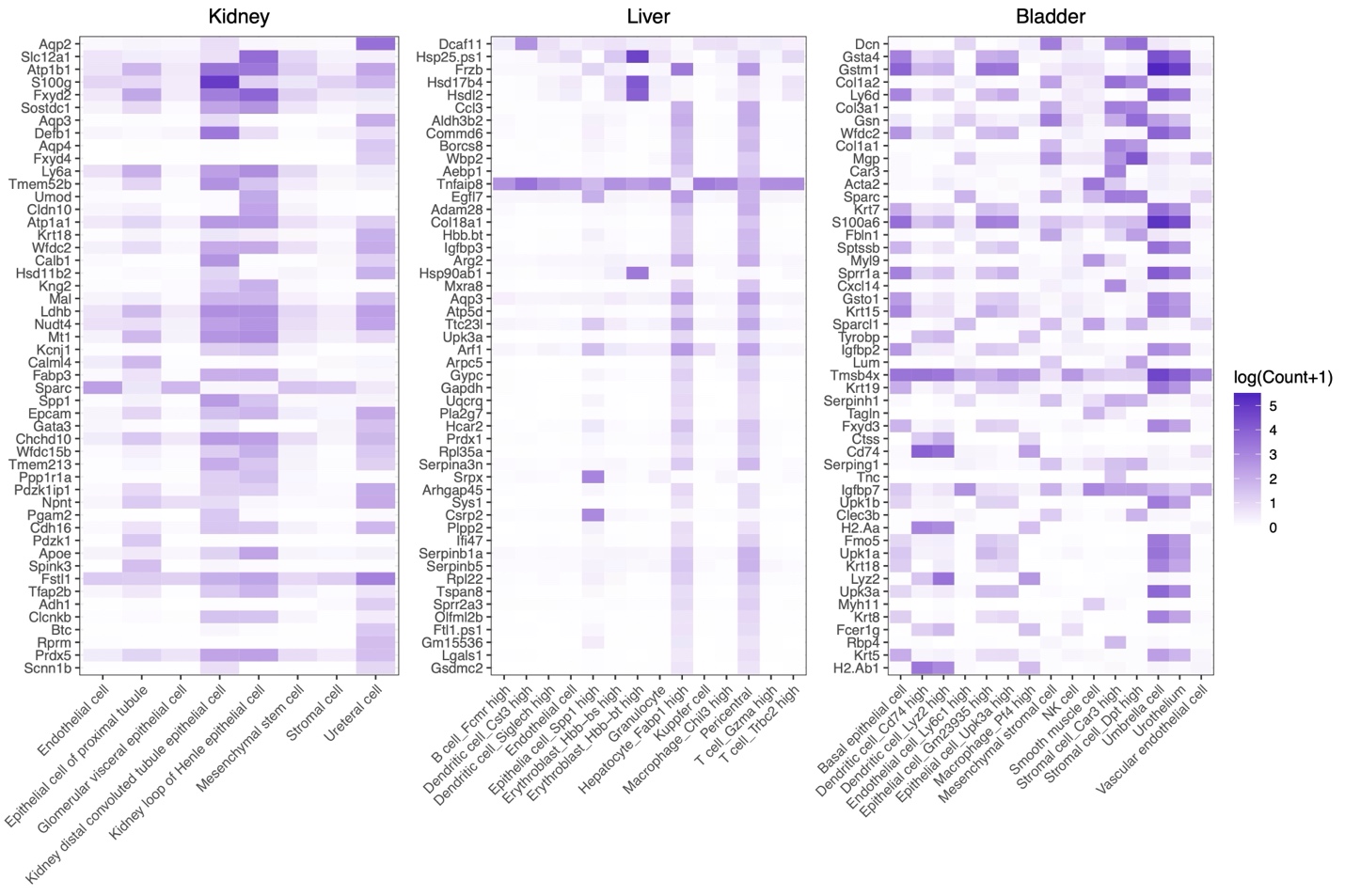


**Supplementary Figure S12.** Heatmap of gene expression of the top 50 genes generated by FEAST for the real scRNA-seq dataset.

**
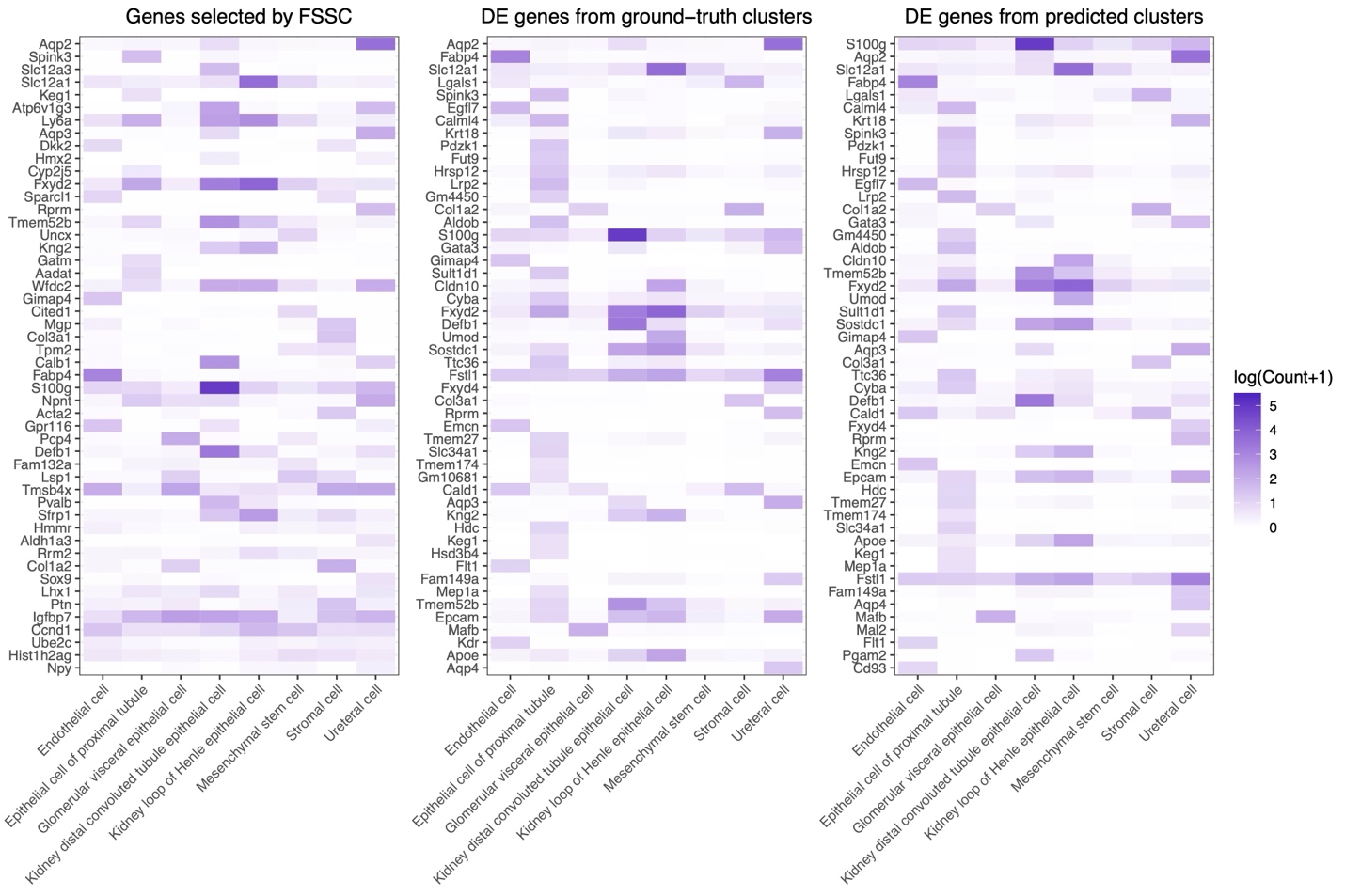
**

**Supplementary Figure S13.** Heatmap of gene expression of the top 50 genes generated by FSSC and from the DE analysis of Kidney dataset.

**
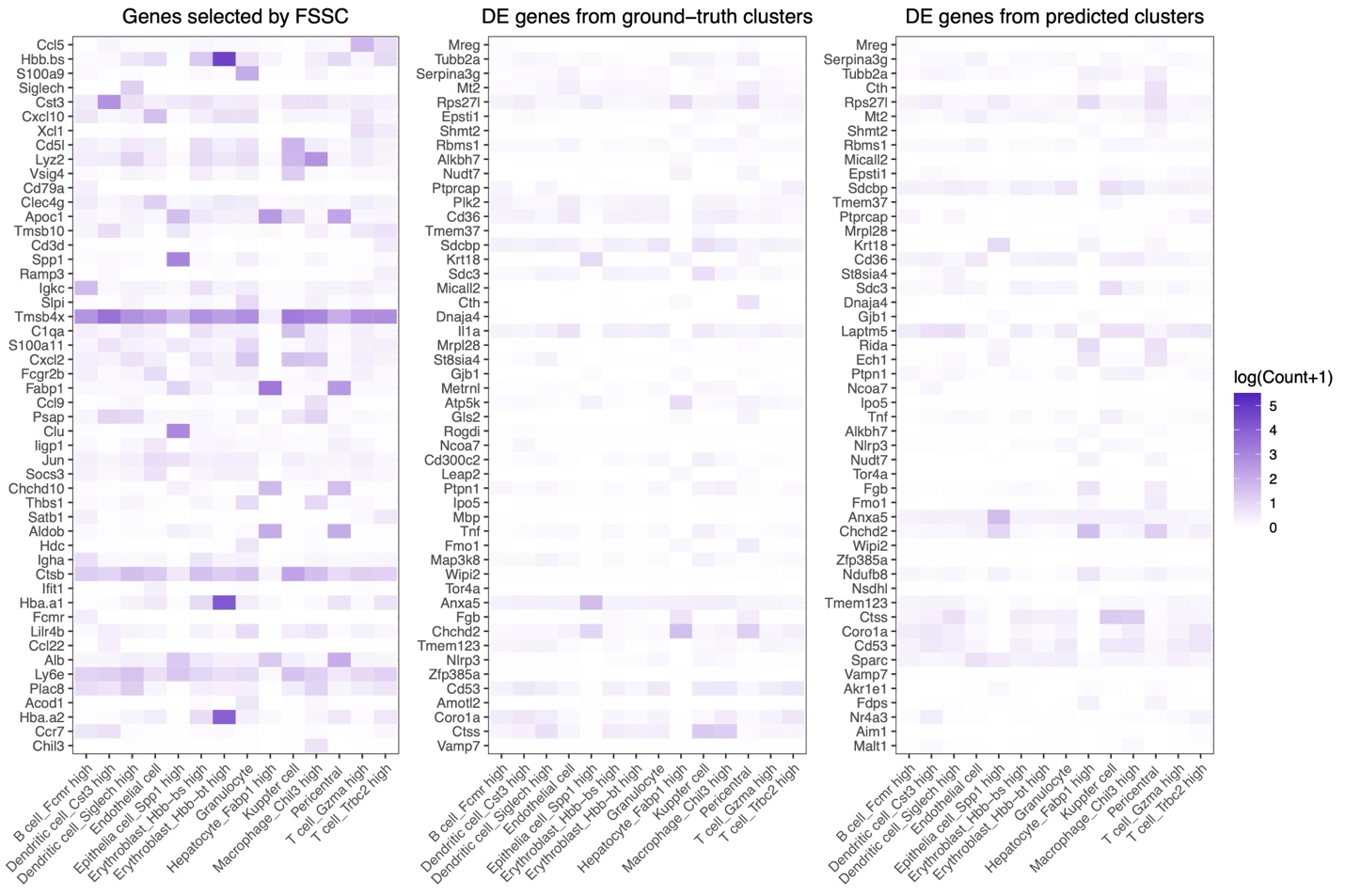
**

**Supplementary Figure S14.** Heatmap of gene expression of the top 50 genes generated by FSSC and from the DE analysis of Liver dataset.

**
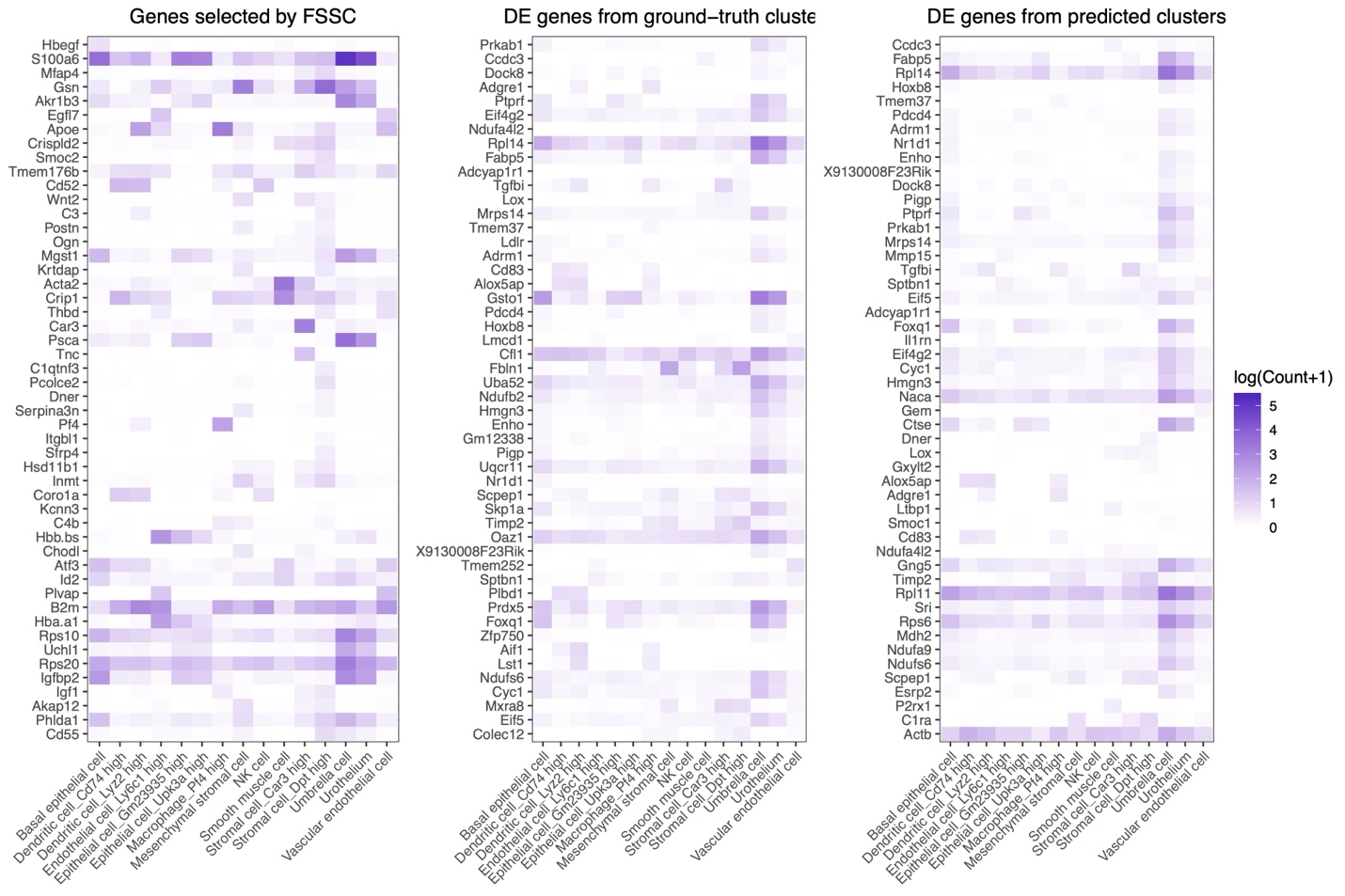
**

**Supplementary Figure S15.** Heatmap of gene expression of the top 50 genes generated by FSSC and from the DE analysis of Bladder dataset.


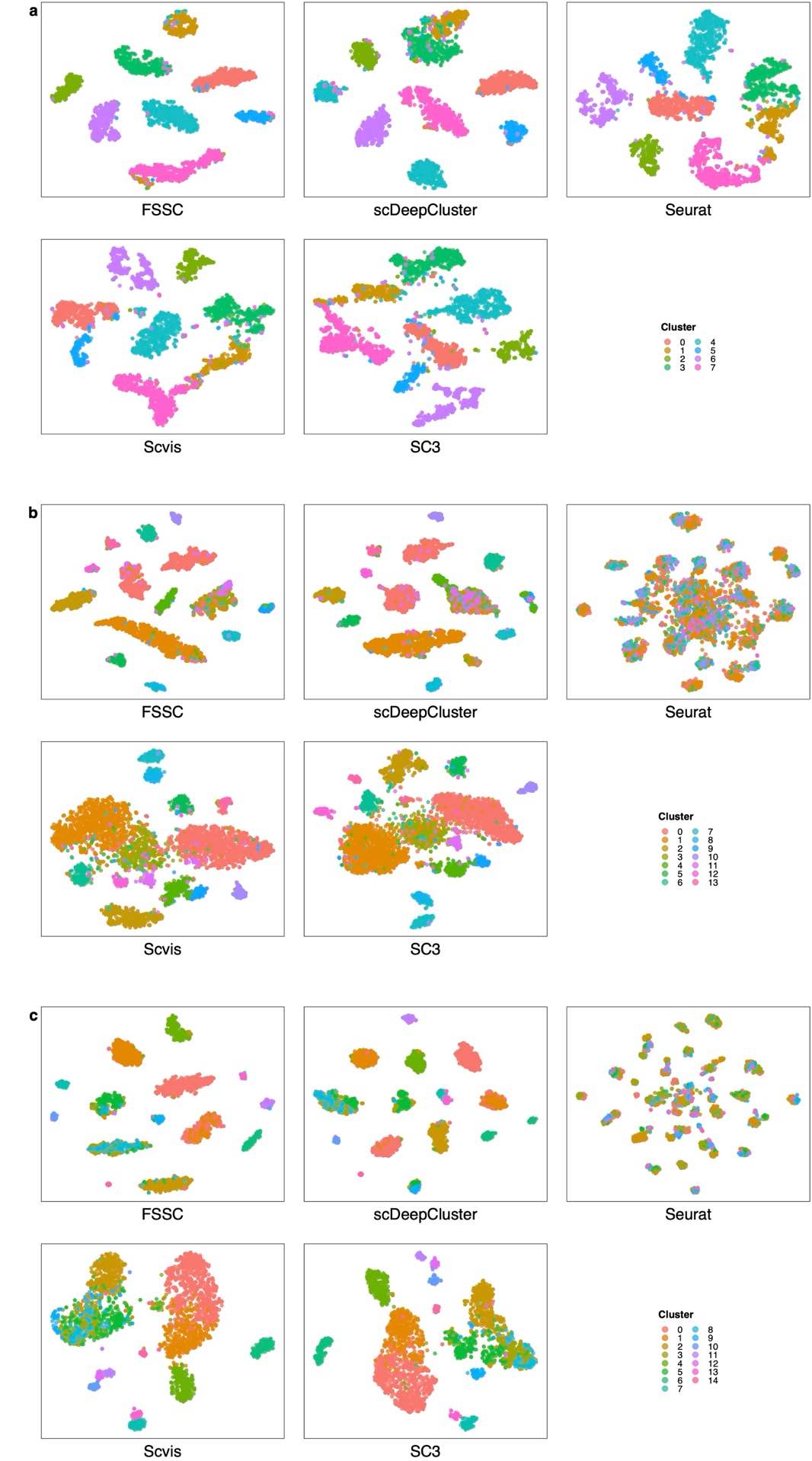


**Supplementary Figure S16.** 2D visualization of embedding. (**a**) Kidney. (**b**) Liver (**c**) Bladder

**Supplementary Note 1. Read count data preprocessing and transformation**

Following the methods of scDeepCluster (Tian, Wan, et al. 2019), we applied the Python package SCANPY (Wolf, Angerer and Theis 2018) to preprocess the raw scRNA-seq read count data. First, we filter out genes with no count in any cell. Next, we calculate the size factors and normalize the read counts by the library size, such that the total counts are the same across cells. Let $s_{i}$ represent the library size of cell $i$, then the size factor of cell $i$ is calculated as $s_{i}/median\left( s \right)$. Then, we take the log transformation and scale the read counts to generate count values with unit variance and zero mean. We use this transformed read count matrix as the input for the denoising ZINB model-based autoencoder and utilize the raw count matrix to calculate the ZINB loss.

**Supplementary Note 2. Model Implementation**

FSSC is implemented in Python 3 (version 3.8.16) using PyTorch (version 1.13.1) (Paszke, et al. 2019). The sizes of hidden layers in the ZINB-based auto-encoder are set to be (256, 64, 32, 64, 256), where the size of bottleneck layer is 32. The standard deviation of Gaussian random noise is 2.5. Adam (Kingma and Ba 2015) with AMSGrad (Reddi, Kale and Kumar 2018) and Adadelta (Zeiler 2012) are applied for pretraining stage and clustering stage, respectively. The parameters of Adam optimizer are set with initial learning rate $lr=0.001, \beta_{1}=0.9$, and $\beta_{2}=0.999$ and parameters of Adadelta optimizer are set to be of $lr=1.0$ and $rho=0.95$. The weight of clustering loss $\gamma$ is set to 1. The batch size for pretraining and clustering is 256. We pretrained the autoencoder 300 epochs. The parameters for gene selection are set as follows: penalty multiplier $\epsilon=1.02$, number of selected feature multiplier

$\omega=0.95$, minimum number of selected features $\xi=32$, which is the same as the bottleneck layer’s size, penalty parameter initial vale $\lambda_{Init}=0.001$. The convergence threshold for clustering stage is 0.1% of the changed clustering labels per epoch. All experiments are conducted on NVIDIA V100 SMX2 GPU.

**Supplementary Note 3. Summary of the implementation of competing methods**

scDeepCluster (Tian, Wan, et al. 2019), Seurat (Stuart, et al. 2019), SC3 (Kiselev, et al. 2017), Scvis (Ding, Condon and Shah 2018)+k-means, FSCseq (Lim, Rashid and Ibrahim 2021), RZiMM-NB (Mi, et al. 2021), NBDrop (Andrews and Hemberg 2019) and FEAST (Su, Yu and Wu 2021) are used as competing methods. scDeepCluster, Seurat, SC3 and Scvis+k-means generate clustering results, while FSCseq and RZiMM-NB can deliver both clustering and feature selection results. Seurat, NBDrop and FEAST provide feature selection results. FSCseq generates binary results for each feature to identify whether they are selected or not. RZiMM-NB, Seurat, NBDrop and FEAST provide the gene importance rank for all genes, but additional threshold is needed to determine the selected genes. For NBDrop, we set the significant threshold to 0.05 as the threshold.

SCDeepCluster (<https://github.com/ttgump/scDeepCluster_pytorch>) is a deep learning based clustering method for scRNA-seq data. It applies an autoencoder and performs clustering on the latent embedding space. We use the parameters as default settings.

SC3 (<https://bioconductor.org/packages/release/bioc/html/SC3.html>) first calculates three different distances matrices, Euclidean, Pearson and Spearman metrics, and combine the results of spectral clustering on distance matrices to produce clustering results. We use the parameters as default settings.

Seurat (<http://satijalab.org/seurat/>) first constructs the Shared Nearest Neighbor (SNN) for all the samples, and identifies cluster assignments via modularity optimization-based clustering algorithm. We tune the parameter “resolution” so that the number of predicted clusters equals to the number of the ground truth groups. Moreover, we use the function “FindVariableFeatures” for feature selection.

Scvis (<https://github.com/shahcompbio/scvis>) is a deep generative dimensionality reduction model for scRNA-seq data. We perform k-means clustering on their output to obtain the final clustering labels. We set the bottleneck layer’s size to 8 and use other parameters as default settings.

FSCseq (<https://github.com/DavidKLim/FSCseq>) applies a penalized mixture Negative Binomial model to perform clustering and feature selection. We set the parameter “med_filt” and “MAD_filt” to 0, and calculate the library size on adding 1 to the count matrix. We use other parameters as default settings.

RZiMM (<https://github.com/SkadiEye/RZiMM>) implements several penalized mixture Zero-Inflated models to perform clustering and feature selection for scRNA-seq data. We use the Zero-Inflated Negative Binomial model RZiMM-NB as benchmarks. We use the parameters as default settings.

NBDrop (<https://github.com/tallulandrews/M3Drop>) is a dropout-based feature selection method. Genes with high dropout rates are identified as features. We set the significant threshold qval.thresh=0.05, and other parameters are set as default settings.

FEAST (<https://github.com/suke18/FEAST>) performs F-test based on the consensus clusters results and ranks the feature significance according the F-statistics. We set “thre = 0”, and other parameters are set as default settings.

**Supplementary Note 4. Detailed description of the simulation settings**

Simulated data are generated by the R package Splatter (Zappia, Phipson and Oshlack 2017). The R function splatSimulate is used to simulate the scRNA-seq count data. We simulated four cell groups, 4000 cells of 10000 genes. We then sampled 1000 of the 10000 genes to generate a $4000\times1000$ raw count matrix in the following way. We divide the 10000 genes into discriminatory $G_{d}$ and nondiscriminatory genes $G_{nd}$, where nondiscriminatory genes are genes with de factors values ($DEFacGroup$ value generated by the Splatter) equal to 1 for all four groups, and discriminatory genes, otherwise. We then selected a subset of $G_{d}$ by filtering the genes with $def\_min<|DEFacGroup| \leq def\_max$, and applied R package glmnet (Friedman et al. 2010) to remove highly correlated genes in the selected subsets by training a multinomial classification model with group-lasso penalty, where the model inputs are the selected gene subsets and the outputs are the ground truth cell assignments. Let $r_{dg}$ be the proportion of discriminatory genes among 1000 genes. We adjusted the lasso penalty to keep $100\dot{0}\times r_{dg}$ discriminatory genes derived from the glmnet, sampled $100\dot{0}\times{(1-r}_{dg})$ nondiscriminatory genes from $G_{nd}$ and concatenated them to generate the final simulation dataset. We repeated all experiments 10 times with different random seeds under the same setting.

To simulation dataset with various dropout rates, we set the parameter $dropout. shape=-1$, $de.prob=0.2$, $de.facScale=0.3$ and vary $dropout.mid$ in (0, 0.5, 1) (the corresponding dropout rates are 25.8 ± 0.5%, 34.3 ± 0.4%, and 43.6 ± 0.7%). The other parameters are set

to default values. Moreover, we set $r_{dg}=0.1$, ${def}_{min}=2^{0.6}$, ${def}_{max}=2^{0.7}$.

To simulate datasets with various signal strengths, we set the parameter $dropout. shape=-1$, $de.prob=0.2$, $de.facScale=0.3$ and $dropout.mid=1$. The other parameters are set

to default values. We also set $r_{dg}=0.1$, and vary the (${def}_{min}, {def}_{max})$ pair in $(2^{0.7},2^{0.8})$, $(2^{0.6},2^{0.7})$ and $(2^{0.5},2^{0.6})$ to generate datasets with high, median and low signal strength.

To simulate datasets with various ratio of discriminatory genes, we set the parameter $dropout. shape=-1$, $de.prob=0.2$, $de.facScale=0.3$ and $dropout.mid=1$. The other parameters are set to default values. We vary the pair ($r_{dg}, {def}_{min}, {def}_{max})$ in $(0.05, 2^{1}, 2^{1.15})$, $(0.1, 2^{0.6}, 2^{0.7})$ and $(0.15, 2^{0.5}, 2^{0.6})$ to generate datasets with similar signal strength but with discriminatory genes ratio changing from 0.05, 0.1, to 0.15.

To simulate datasets without dropout event with different signal strengths, we follow the aforementioned simulation steps but generate dataset with total 600 genes. We set the parameter $dropout. shape=-1$, $de.prob=0.2$, $de.facScale=0.3$, $dropout.mid=1$ and $dropout.type="none"$. The other parameters are set to default values. We also set $r_{dg}=0.1$, and vary the (${def}_{min}, {def}_{max})$ pair in $(2^{0.5},2^{0.6})$ and $(2^{0.6},2^{0.7})$ and $\left( 2^{0.7},2^{0.8} \right)$ to generate dataset with High, median and low signal strength.

**Supplementary Note 5. Detailed description of the real scRNA-seq data**

The Kidney dataset was provided by the authors (Adam, Potter and Potter 2017). The authors analyzed the data and identified the cell types. We download the expression matrix from <https://github.com/xuebaliang/scziDesk>. The preprocessed data contains 3660 cells from 8 groups.

The Liver and Bladder datasets were provided by the authors (Han, et al. 2018). The authors identified the cell types. We downloaded the batch removed expression matrix from <https://figshare.com/s/865e694ad06d5857db4b>, and selected cells from Liver and bladder tissue, respectively. We follow (Chazarra-Gil, et al. 2021) to preprocessing the data by removing cells expressing <250 genes, genes expressed in <50 cells, cell types representing <1% of total cell population in a tissue. The Liver dataset contains 4553 cells from 14 groups and Bladder dataset contains 2733 cells from 15 groups.

**Supplementary Note 6. Definition of Cluster-specific score**

We apply an entropy-based evaluation method, the cluster-specific score (Cabili, et al. 2011) (Tian, Wei, et al. 2018), to quantify the gene selection results.

For each gene $g$, let $x_{ik}$ denote the raw count data of cell $i$ with ground truth cell type $k$, where $k\in[1,2, \cdots, K]$, $K$ is the number of cell types, and $i\in[1,2, \cdots, n_{ik}]$, $n_{ik}$ is the number of cells having type $k$. We first calculate the average of the log-transformed gene expression across cell types, denoted by $\bar{x}_{k}=\sum_{i=1}^{n_{ik}} \log_{2} \left( x_{ik}+1 \right)/n_{ik}$. If one gene is perfectly cluster-specific, then $\bar{x}_{k}$ should be zero in all types except one. Next, we calculate the normalized gene expression in various cell types, $E=(e_{1}, e_{2}, \cdots, e_{K})$, where $e_{k}=\bar{x}_{k}/\sum_{i=1}^{K} \bar{x}_{k}$ represents the proportion of total expression in the $k$th cell type. Then, we can define the ideal expression profile in each cell type, $E^{1}=\left( 1,0,\cdots, 0 \right), E^{2}=\left( 0,1,\cdots, 0 \right),\cdots,E^{K}=\left( 0,0,\cdots, 1 \right)$. $E^{k}$ corresponds to an extreme case where the gene is only expressed in the $k$th cell type. Finally, we calculate the distance between observed gene expression profile $E$ and the ideal expression profile $E^{k}$ via the Jensen-Shannon divergence, $JS\left( EE^{k} \right)=H\left( \frac{E+E^{k}}{2} \right)-\frac{H\left( E \right)+H\left( E^{k} \right)}{2}$, where $H$ is the Shannon entropy. The cluster-specific score is defined as $S\left( E \right)=1-\min_{k} \sqrt{\mathrm{JS}\left( \mathrm{EE}^{k} \right)}$. A larger score indicates a greater cluster type specificity, and a score of 1 means the gene is expressed in only one cell type.

**Supplementary Note 7. Algorithms**

**
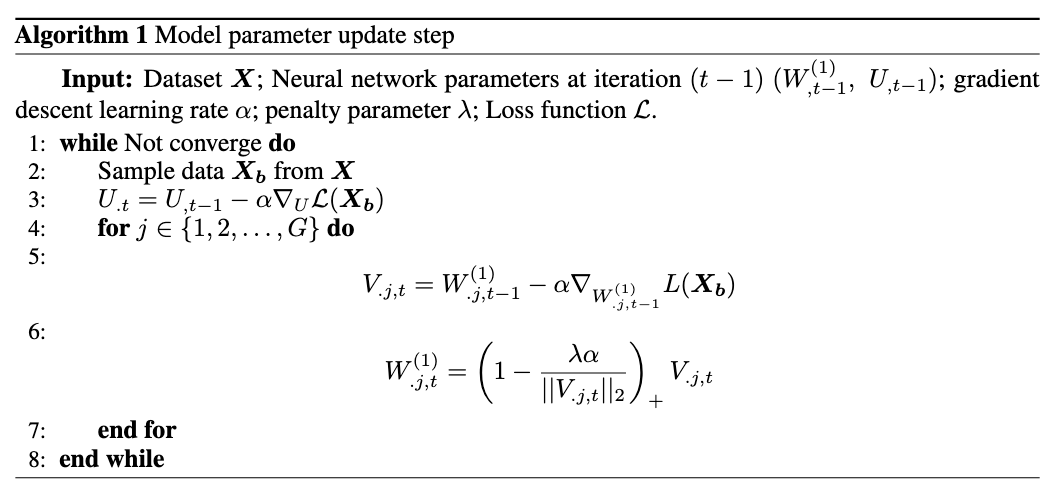
**

**
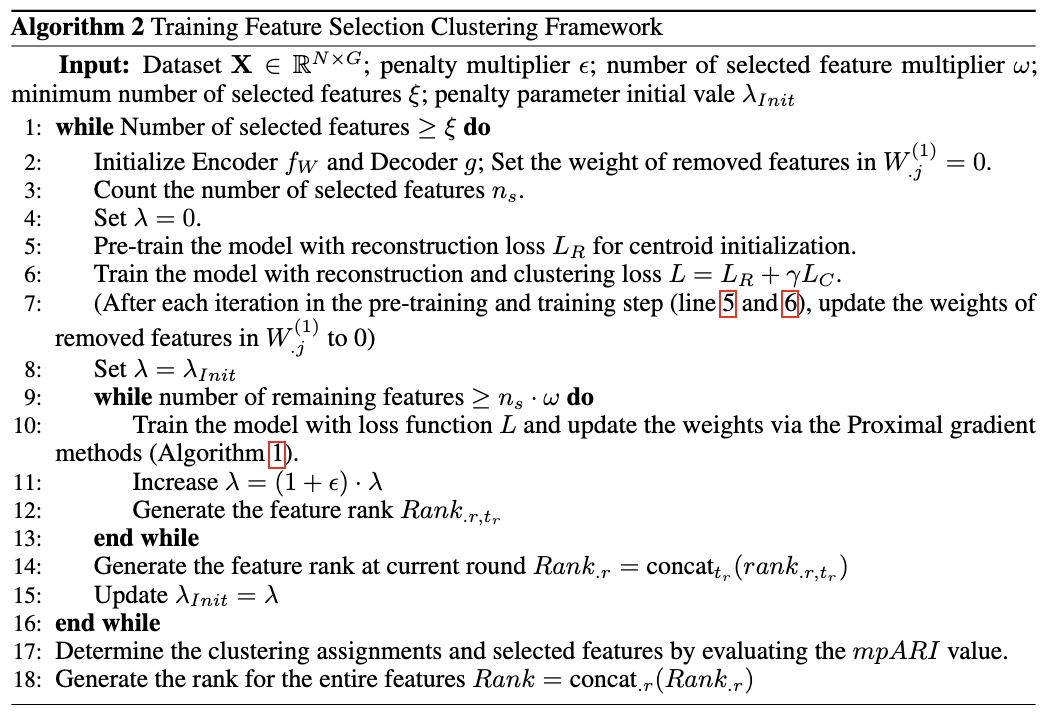
**

**Supplementary Note 8. Tables**

Table 1: Summary of three real scRNA-seq datasets

| **Dataset** | **Cell Number** | **No. of genes** | **No. of groups** |
| --- | --- | --- | --- |
| Kidney | 3360 | 23797 | 8 |
| Liver | 4553 | 7816 | 14 |
| Bladder | 2733 | 9578 | 15 |

Table 2: Number of selected Features

|  | **Kidney** | **Liver** | **Bladder** |
| --- | --- | --- | --- |
| FSSC | 250 | 373 | 756 |
| FSCseq | 1906 | 1775 | 1890 |
| NBDrop | 650 | 178 | 543 |

Table3: Number of Overlap between FSSC-selected genes and DE genes identified by MAST (Finak et al., 2015)

| **Dataset** | **Overlap with Ground Truth DE** | **Overlap with FSSC-label DE** |
| --- | --- | --- |
| Kidney | 36 | 37 |
| Liver | 45 | 50 |
| Bladder | 28 | 34 |

Table4: Comparison of selected genes and their overlap with known marker genes (Adam, Potter and Potter 2017) across different feature selection methods on the kidney dataset.

|  | **Overlap with Marker Genes** | **Recall (%)** |
| --- | --- | --- |
| FSSC | Osr2 Lhx1 Jag1 Rbp1 Napsa Ttc36 Kap Gpc3 Mest Alcam Dcn Penk Wnt11 Sox9 Aqp2 Aqp3 Aqp4 | 6.8 |
| FSCseq | Hnf4a Osr2 Lhx1 Jag1 Gpx3 Acaa2 Napsa Ttc36 Slc3a1 Slc23a1 Slc27a2 Kap Aldh1a2 Mest Alcam Zeb2 Dcn Penk Col15a1 Wnt11 Ret Krt23 Etv4 Etv5 Gfra1 Aqp2 Aqp3 Aqp4 Scnn1a Scnn1b Scnn1g Foxi1 Slc26a4 Slc4a1 | 1.8 |
| NBDrop | Lhx1 Rbp1 Acaa2 Napsa Ttc36 Slc23a1 Slc27a2 Kap Gpc3 Mest Dcn Penk Wnt11 Ret Krt23 Sox9 Gfra1 Aqp2 Aqp3 Aqp4 Scnn1a Scnn1b Scnn1g Foxi1 Slc26a4 | 3.8 |

# References

Adam, Mike, Andrew S Potter, and S Steven Potter. 2017. "Psychrophilic proteases dramatically reduce single-cell RNA-seq artifacts: a molecular atlas of kidney development." *Development* 144 (19): 3625-3632.

Andrews, Tallulah S, and Martin Hemberg. 2019. "M3Drop: dropout-based feature selection for scRNASeq." *Bioinformatics* 35 (16): 2865-2867.

Cabili, Moran N, Cole Trapnell, Loyal Goff, Magdalena Koziol, Barbara Tazon-Vega, Aviv Regev, and John L Rinn. 2011. "Integrative annotation of human large intergenic noncoding RNAs reveals global properties and specific subclasses." *Genes Development* 25 (18): 1915-1927.

Chazarra-Gil, Ruben, Stijn van Dongen, Vladimir Yu Kiselev, and Martin Hemberg. 2021. "Flexible comparison of batch correction methods for single-cell RNA-seq using BatchBench." *Nucleic acids research* 49 (7): e42-e42.

Ding, Jiarui, Anne Condon, and Sohrab P Shah. 2018. "Interpretable dimensionality reduction of single cell transcriptome data with deep generative models." *Nature communications* 9 (1): 1-13.

Han, Xiaoping, Renying Wang, Yincong Zhou, Lijiang Fei, Huiyu Sun, Shujing Lai, Assieh Saadatpour, et al. 2018. "Mapping the mouse cell atlas by microwell-seq." *Cell* 172 (5): 1091-1107.

Kingma, Diederik P, and Jimmy Ba. 2015. "Adam: A method for stochastic optimization." *3rd International Conference on Learning Representations (ICLR).*

Kiselev, Vladimir Yu, Kristina Kirschner, Michael T Schaub, Tallulah rews, Yiu, rew, Ch, et al. 2017. "C3: consensus clustering of single-cell RNA-seq data." *Nature methods* 14 (5): 483-486.

Lim, David K, Naim U Rashid, and Joseph G Ibrahim. 2021. "Model-based feature selection and clustering of RNA-seq data for unsupervised subtype discovery." *The annals of applied statistics* 15 (1): 481-508.

Mi, Xinlei, William Bekerman, Peter A Sims, Peter D Canoll, and Jianhua Hu. 2021. "RZiMM-scRNA: A regularized zero-inflated mixture model framework for single-cell RNA-seq data." *arXiv preprint arXiv:2110.12964.*

Paszke, Adam, Sam Gross, Francisco Massa, Adam Lerer, James Bradbury, Gregory Chanan, Trevor Killeen, et al. 2019. "Pytorch: An imperative style, high-performance deep learning library." *Advances in neural information processing systems* 32.

Reddi, Sashank J, Satyen Kale, and Sanjiv Kumar. 2018. "On the convergence of adam and beyond." *International Conference on Learning Representations (ICLR).*

Stuart, Tim, Butler, rew, Paul Hoffman, Christoph Hafemeister, Efthymia Papalexi, William M Mauck III, et al. 2019. "Comprehensive integration of single-cell data." *Cell* 177 (7): 1888-1902.

Su, Kenong, Tianwei Yu, and Hao Wu. 2021. "Accurate feature selection improves single-cell RNA-seq cell clustering." *Briefings in Bioinformatics* 22 (5): bbab034.

Tian, Tian, Ji Wan, Qi Song, and Zhi Wei. 2019. "Clustering single-cell RNA-seq data with a model-based deep learning approach." *Nature Machine Intelligence* 1: 191-198.

Tian, Tian, Zhi Wei, Xiao Chang, Yichuan Liu, Raquel E Gur, Patrick MA Sleiman, and Hakon Hakonarson. 2018. "The long noncoding RNA landscape in amygdala tissues from schizophrenia patients." *EBioMedicine* 34: 171-181.

Wolf, F Alexander, Philipp Angerer, and Fabian J Theis. 2018. "SCANPY: large-scale single-cell gene expression data analysis." *Genome biology* (Genome biology) 19 (1): 1-5.

Zappia, Luke, Belinda Phipson, and Alicia Oshlack. 2017. "Splatter: simulation of single-cell RNA sequencing data." *Genome biology* 18 (1): 174.

Zeiler, Matthew D. 2012. "Adadelta: an adaptive learning rate method." *arXiv preprint arXiv:1212.5701.*

Finak, G., McDavid, A., Yajima, M., Deng, J., Gersuk, V., Shalek, A. K., Slichter, C. K., Miller, H. W., McElrath, M. J., & Prlic, M. (2015). MAST: a flexible statistical framework for assessing transcriptional changes and characterizing heterogeneity in single-cell RNA sequencing data. Genome biology, 16(1), 278.

Adam, M., Potter, A. S., & Potter, S. S. (2017). Psychrophilic proteases dramatically reduce single-cell RNA-seq artifacts: a molecular atlas of kidney development. *Development*, *144*(19), 3625-3632.

Finak, G., McDavid, A., Yajima, M., Deng, J., Gersuk, V., Shalek, A. K., Slichter, C. K., Miller, H. W., McElrath, M. J., & Prlic, M. (2015). MAST: a flexible statistical framework for assessing transcriptional changes and characterizing heterogeneity in single-cell RNA sequencing data. *Genome biology*, *16*(1), 278.
